# Predicting antigen-specific T-cell immunity against Wilms tumor 1 in hematologic cancer

**DOI:** 10.1038/s41375-025-02727-y

**Published:** 2025-08-22

**Authors:** Brittany L. Ford, Emmi Jokinen, Jani Huuhtanen, Sofia Forstén, Jay Klievink, Gabriella Antignani, Oscar Brück, Vincenzo Cerullo, iCAN, Brittany L. Ford, Emmi Jokinen, Gabriella Antignani, Vincenzo Cerullo, Karita Peltonen, Satu Mustjoki, Karita Peltonen, Satu Mustjoki

**Affiliations:** 1 Translational Immunology Research Program (TRIMM), University of Helsinki, Helsinki, Finland; 2 Hematology Research Unit Helsinki, University of Helsinki and Helsinki University Hospital Comprehensive Cancer Center, Helsinki, Finland; 3 iCAN Digital Precision Cancer Medicine Flagship, Helsinki, Finland; 4 Laboratory of Immunovirotherapy, Drug Research Program, Faculty of Pharmacy, University of Helsinki, Helsinki, Finland; 5 Helsinki Institute of Life Science (HiLIFE), University of Helsinki, Helsinki, Finland; 6 Hematoscope Lab, Comprehensive Cancer Center, Helsinki University Hospital, Helsinki, Finland; 7 Department of Oncology, University of Helsinki, Helsinki, Finland; 8 Department of Clinical Chemistry, HUS Diagnostic Center, University of Helsinki and Helsinki University Hospital, Helsinki, Finland; 9 Department of Molecular Medicine and Medical Biotechnology and CEINGE, Naples University 24 Federico II, Naples, Italy

**Keywords:** Translational research, Immunology

## Abstract

Wilms tumor 1 (WT1) is a tumor-associated antigen expressed in solid tumors and hematological malignancies. T-cell immunotherapies targeting WT1 are currently under development. To analyze endogenous T-cell responses against WT1, we trained computational models capable of detecting WT1-specific T-cell responses from T-cell receptor (TCR) sequencing data. We peptide-pulsed healthy donor and acute myeloid leukemia (AML) patient samples with VLDFAPPGA (VLD, WT1_37-45_) and RMFPNAPYL (RMF, WT1_126-134_) peptides, then sequenced the WT1 dextramer-positive CD8 + T-cells with single-cell RNA + TCRαβ sequencing. The TCRGP machine-learning TCR-classification method was trained with epitope-specific and control TCR repertoires, and we obtained AUROC values of 0.74 (VLD) and 0.75 (RMF), allowing reliable identification of WT1-specific T-cells. In bulk TCRβ sequenced patient samples (AML n = 21, chronic myeloid leukemia (CML) n = 26, and myelodysplastic syndrome n = 25), the median WT1-specific T-cell abundance was similar to healthy controls, but their VLD and RMF-specific TCR repertoires exhibited higher clonality with two patients presenting up to 13% of WT1-specific T-cells. ScRNA+TCRαβ sequencing of AML bone marrow T-cells revealed that WT1-specific T-cells predominantly exhibit an effector or terminal effector memory phenotype. In conclusion, our novel computational models enable large-scale WT1-specific T-cell identification from TCR sequencing datasets and leukemia-antigen-specific immune response monitoring.

## Introduction

Wilms tumor 1 (WT1) is a zinc finger transcription factor, typically expressed at low levels in hematopoietic stem cells and specialized cells of the urogenital system during development [1, 2]. During leukemogenesis an increased WT1 expression of up to 10 - 1000-fold has been observed in leukemic CD34+ cells and high expression levels correlate with aggressive disease [3]. WT1-specific T-cells have previously been minutely observed in both healthy controls and patients with hematological cancers [4]. As T-cells that bind to self-peptide-MHC complexes with high affinity are usually negatively selected within the thymus [5, 6], endogenous TCRs to WT1 likely result from chronic WT1 exposure enhancing auto-reactive T-cells with low avidity for WT1 epitopes [6–8]. Yet, WT1 presents an ideal target [9, 10] for developing autologous epitope-specific T-cell therapies and monitoring disease progression in hematologic malignancies. In AML patients, donor-derived CD8 + T-cells modified to express WT1-specific TCRs have already been tested to prevent relapse post hematopoietic stem cell transplantation (HSCT), and promising initial responses have been obtained [11, 12].

The avidity and diversity of peptides that TCRs recognize are typically driven by their complementarity-determining region 3 (CDR3). This sequence, along with the variable, joining, and diversity genes from α- and β-chains can form up to 10^15^–10^20^ unique TCRαβ -pairs [13], which can recognize a vast number of epitopes [13–15]. Advances in TCR sequencing have enabled the development of computational methods to characterize conserved TCR motifs [16]. For example, GLIPH [17] enables the grouping of TCR-specificities based on CDR3 sequences in silico. However, a lack of antigen-specific training data has prevented computational tool development for many endogenous antigens, such as for WT1, and studying antigen-specific T-cell responses at scale.

**Fig. 1:**
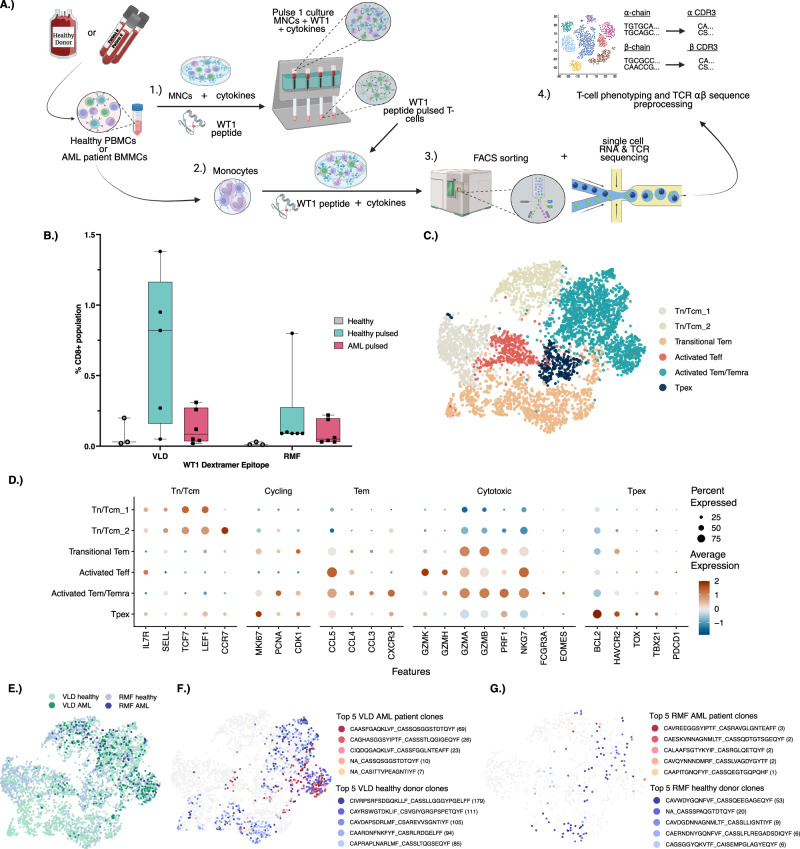
Peptide pulsation of WT1 and its specific T-cell phenotypes. **A** Graphical summary of the experimental pipeline for WT1-specific TCR enrichment. In this study, peripheral blood mononuclear cells (PBMCs) or bone marrow mononuclear cells (BMMCs) were stimulated (pulsed) with Wilms tumor 1 (WT1) peptides to enrich CD8 + T-cells specific to these epitopes. The enriched CD8 + T-cells were then sorted using fluorescence-activated cell sorting (FACS) based on WT1-dextramer binding and subjected to 10X Genomics sequencing for RNA and T-cell receptor (TCR) sequences. These sequences were used to train predictive models to identify WT1-specific CD8 + T-cells across different patient cohorts. **B** Percentages of WT1-specific CD8 + T-cells from healthy donor PBMCs before peptide pulsation (Healthy), after 2 weeks of peptide pulsation (Healthy pulsed), and from AML patient BMMCs after 2 weeks of peptide pulsation (AML pulsed). Data is shown for CD8 + T-cells specific to WT1 epitopes VLDFAPPGA (VLD) and RMFPNAPYL (RMF). **C** Uniform manifold approximation and projection (UMAP) representation of WT1-pulsed, WT1-dextramer sorted CD8 + T-cells, sequenced for both RNA and TCRαβ. Manually annotated CD8 + T-cell clusters include: naïve central memory (Tn/Tcm_1 or Tn/Tcm_2), transitional effector memory (Transitional Tem), activated effector (Activated Teff), activated effector memory terminally differentiated effector memory (Activated Tem/Temra), and precursor exhausted memory (Tpex). **D** Plot shows the scaled average expression and proportion of the cells within each cluster that express cluster-defining markers. **E** UMAP visualization of CD8 + WT1-specific T-cells, colored by donor type (healthy or AML patient) and WT1-epitope (VLD or RMF). **F**, **G** UMAP displays the five most abundant (Top 5) TCR clonotypes from AML patients and healthy donors for VLD (**F**) and RMF (**G**).

To overcome this, we utilized peripheral blood mononuclear cells (PBMCs) from healthy donors (n = 6) and acute myeloid leukemia (AML) patient bone marrow mononuclear cells (BMMCs) (n = 6) to enhance WT1-specific T-cell responses through peptide-pulsing (study flow shown in Fig. 1A). With scRNA+TCRαβ sequencing we were able to analyze WT1-specific TCRs and utilize them together with existing TCR datasets [18, 19] to train TCRGP (where TCR refers to T-cell receptors and GP to Gaussian processes) [20] machine-learning classifiers to predict WT1-specific T-cells. Using these novel models, we analyzed bulk TCRβ and scRNA+TCRαβ-seq datasets from AML, CML, and MDS patients to quantify WT1-specific T-cell frequencies and phenotypic characteristics. Our results confirm that WT1-specific T-cells can be detected in both peripheral blood and bone marrow samples with high interindividual variation.

## Materials and Methods

### Mononuclear cell samples for WT1 peptide pulsing

PBMCs from 6 HLA-A*02:01 homozygous, healthy controls and BMMCs from 6 newly diagnosed, untreated AML patients were separated with Ficoll-Paque centrifugation (GE Healthcare, Chicago, Illinois, USA) and used for antigen pulsing procedures. All mononuclear cells (MNCs) were cultured in RPMI 1640 supplemented with 2mM L-glutamine, 10% FBS, and 1 mM sodium pyruvate (referred to as R10) (Gibco, Paisley, Scotland).

This study complied with the Declaration of Helsinki, including informed consent, and was approved by the HUS ethics committee (DNRO 303/13/03/01/2011). Healthy control PBMC samples were obtained from Finnish Red Cross Blood Service (13-2022).

### WT1 antigen pulsation procedure

The antigen pulsation protocol was adapted from Schmied et al. [4]. Briefly, whole PBMCs from healthy donors or BMMCs from AML patients were stimulated separately with 6 uM VLDFAPPGA (VLD) or 6 uM RMFPNAPYL (RMF) WT1 peptides (JPT, Berlin, Germany) for 24 - 36 h in the presence of anti-CD28 antibody and β2-microglobulin (detailed in the Supplementary methods), not as a pool. Then, CD8 + T-cells were isolated using anti-CD8 beads (Miltenyi Biotech, Bergisch Gladbach, Germany) and cultured in R10 with 25 ng/mL IL-21 (PeproTech, Cranbury, NJ, USA) for 48 h to enhance T-cell priming and support early T-cell survival. Next, 10 ng/mL IL-15 (PeproTech) supplementation was added for 48 h to enhance T-cell expansion. Lastly, cells were split and incubated in IL-15-supplemented R10 for 48 h.

Meanwhile, monocytes from the corresponding frozen PBMCs or BMMCs were isolated using anti-CD14 beads **(**Miltenyi Biotech) and peptide-loaded for 3-4 h. Then the expanded CD8 + T-cells were co-cultured with peptide-loaded CD14+ cells for 48 h, followed by another split and incubation in IL-15 supplemented R10 for 48 h.

### WT1 dextramer staining and fluorescence-activated cell sorting

Dead cells were removed from live, WT1-stimulated cells via Miltenyi Biotech bead kit, diluted, and stained with VLDFAPPGA or RMFPNAPYL Dextramer per manufacturer’s instructions (Immudex, Copenhagen, Denmark). Antibodies (*αCD14-PE-Cy7, αCD3- PerCP-Cy5.5, αCD4-BV510, αCD8-APC-H7*) for CD8 + T-cell gating were added and dextramer-positive cells were sorted using BD Influx. VLD and RMF positive cell gates are shown in Supplementary Fig. 1.

### Single-cell RNA + TCRαβ sequencing, and data processing

Single-cell gene expression profiles of WT1-specific, FACS-sorted CD8 + T-cells were studied using 10X Genomics Chromium Single Cell 5’ Gene expression with Feature Barcoding technology platform (TCR sequences in Supplemental Tables 1–4). The Chromium Single Cell 5’RNAseq run and library preparation used Chromium Next GEM Single Cell 5’ Immune Profiling with Feature Barcoding technology version 2 chemistry. The Sample libraries were sequenced on the Illumina NovaSeq 6000 system. Data processing and analysis used 10X Genomics Cell Ranger v7.2.0 pipelines. VDJ-clonotypes were generated per sample via Cell Ranger and VDJ FASTQs for the fill library.

The R package Seurat (v5.1) [21] was used for further scRNA-seq data processing. Cell filters for selecting TCRs for TCRGP model training included: mitochondrial gene counts>10%, UMI counts<1500 or > 70,000, detected gene counts < 800 or > 7000 (see Supplemental Fig. 2), and cells without TCRβ or with multiple TCRβs. After log-normalization, highly variable genes were calculated by “FindVariableFeatures”, using ‘vst’ selection, where TCR genes were removed. Cell cycle phases were scored by “CellCycleScoring”. Phase effects were regressed out during data scaling. Harmony [22] was used for the batch correction of four sequencing runs: VLD Healthy pulsed, VLD AML patient pulsed, RMF Healthy pulsed, and RMF AML patient pulsed. Clusters were defined based on the top 20 principal components using “FindNeighbors” and “FindClusters” functions.

### TCR clustering with GLIPH2

GLIPH2 [23] was used to identify TCR-sequence motifs enriched in the VLD- or RMF-specific TCR-repertoires, compared to the human CD8 v2 reference of 573 211 unselected naïve CD8 T-cells provided with the GLIPH2 package (http://50.255.35.37:8080/tools). Default parameters were applied, except adjustments to local_min_pvalue (set to 0.0001), kmer_min_depth (set to 5), and local_min_OVE (set to 1000) to obtain a more restricted set of TCRs. GLIPH2-defined clusters were visualized using the Python package NetworkX v. 3.2.1 with GraphViz neato layout.

### Epitope-specific TCR repertoires for WT1 pulsed samples

From six healthy and six AML patient donors with HLA-A*02:01, we obtained 6833 and 666 VLD-specific TCRs, respectively; we similarly obtained 1138 and 130 RMF-specific TCRs. 33 TCRs found in both VLD and RMF repertoires were removed to ensure as clean training data for the TCRGP model training as possible similarly to Gielis et al.[24]. A total of 7499 VLD- and 1268 RMF-specific TCRs were combined with 261 VLD- and 101 RMF-specific TCRs by Gielis et al. [19, 24](Gielis-healthy). Then GLIPH2 [23]was applied to generate representative repertoires for each epitope and to select for TCRs containing sequence similarity. All TCRs contained TCRβ-sequences, only incorporating TCRα-sequences when available. This yielded 1369 TCRs for VLD (520 from healthy, 102 from AML, and 747 from Gielis-healthy) and 362 TCRs for RMF (282 from healthy, 33 from AML, and 47 from Gielis-healthy). Among VLD-specific TCRs, one TCR was shared between healthy and Gielis-healthy; otherwise, there was no overlap. Lastly, TCRs with unique CDR3β-sequences were selected, resulting in final repertoires of 1366 VLD-specific and 360 RMF-specific TCRs, listed in Supplemental Tables 5 and 6.

### TCRGP models for epitope-specificity prediction

TCRGP is a Gaussian process-based machine-learning method for predicting the epitope-specificity of TCRs [20]. To train TCRGP models per epitope, we used epitope-specific and control TCRs (non-WT1 or viral-targeting TCRs from VDJdb) in a 1:10 ratio [18, 25, 26]. Epitope-specific TCRs for VLD and RMF were selected using GLIPH2 by enriching for TCRs containing sequence similarity, as described above.

As a control to WT1-specific TCRs, we also trained TCRGP models specific to viral antigens and these included cytomegalovirus (CMV) epitope NLVPMVATV, influenza A virus (IAV) epitope GILGFVFTL, Epstein-Barr virus (EBV) epitope GLCTLVAML, severe acute respiratory syndrome coronavirus 2 (SARS-CoV-2) epitope YLQPRTFLL, and melanoma epitope ELAGIGILTV. These models were trained on publicly available TCRs with a confidence score ≥ 1 from VDJdb [26]. All TCRGP models were trained using TCRβ chain CDRs, defined by CDR3β and Vβ-gene. Model evaluation utilized stratified 10-fold cross-validation, and for each model, a prediction threshold corresponding to a false positive rate (FPR) of 0.01 was used. The area under receiver operating characteristic (AUROC) and area under precision-recall (AUPR) curves for each model were computed by the R-package precrec [27] version 0.14, shown in Supplementary Fig. 3.

### TCRβ and scRNA+TCRαβ sequencing cohorts

Healthy control and patient cohorts are listed in Table 1, with individual demographic and clinical details in Supplementary Tables 7-13. The healthy cohort, HC-PB, contains 20 CMV-negative and 20 CMV-positive donors randomly selected from the Emerson et al. cohort [28]. The bulk TCRβ cohorts include 21 AML bone marrow samples (AML-BM) [29], 15 MDS patient samples, 4 myelodysplastic/myeloproliferative neoplasm (MDS-MPN) samples, 5 chronic myelomonocytic leukemia (CMML) samples, and 1 MDS suspicion sample (MDS-BM, unpublished), 14 CML bone marrow samples, and 12 CML blood samples [30]. All bulk TCRβ cohorts were down-sampled to 40,000 reads. The single-cell RNA + TCRαβ cohorts include bone marrow samples from six AML and one MDS patient (AML-BM-SC, unpublished), and six blood samples from CML patients sampled in remission, 3-14 years post-diagnosis upon tyrosine kinase inhibitor therapy discontinuation (CML-PB-SC) [31]. The processing of the scRNA + TCR*αβ -*sequencing data for AML-BM-SC and CML-PB-SC is described in Supplemental Materials and Methods.

**Table 1:** Cohort summary.

Cohort	Number of patients	Sequence type	Average TCR count	Average TCR count downsampled	Source DOI
AML-BM	21	BM TCRβ	28,262	19,390	10.1182/bloodadvances.2019000792
MDS-BM	25	BM TCRβ	17,097	17,061	unpublished
CML-BM	14	BM TCRβ	77,345	20,425	10.1038/s41375-023-02074-w
CML-PB	12	PB TCRβ	27,640	24,681	10.1038/s41375-023-02074-w
HC-PB	40	PB TCRβ	424,997	40,000	10.1038/ng.3822
AML-SC-BM	7	BM scRNA+TCRαβ	3667	3667	unpublished
CML-SC-PB	6	BM scRNA+TCRαβ	3631	3631	10.1038/s41375-023-02074-w

*TCR* T cell receptor, *AML* acute myeloid leukemia, *BM* bone marrow, *MDS* myelodysplastic syndrome, *CML* chronic myeloid leukemia, *PB* peripheral blood, *HC* healthy control, *SC* single cell, *DOI* digital object identifier.

### Cohort visualizations

Seqlogos [32], CDR3b clonality treemaps [33], and Venn diagram [34] visualizations were generated using the R packages ggseqlogo version 0.2, treemap version 2.4-4 and VennDiagram version 1.7.3.

### HLA class I genotyping and WT1 epitope binding prediction

OptiType [35] was used to predict patient HLA-class-I (HLA-I) genotypes for AML and CML single-cell cohorts. NetMHCpan 4.1b [36] was used to predict VLD and RMF binding-capacity for patient-specific HLA-I alleles. HLA-I binder predictions, shown in Supplementary Tables 12-13, are marked as strong (HLA in top 0.5%) or weak (HLA in top 2%) based on binding rank. HLA class I allele sequence binding motifs were adopted from NetMHCpan 4.1 Motif Viewer tool.

### Statistical analyses

One-sided Fisher’s exact tests with Benjamini–Hochberg correction were used to assess phenotypic enrichment of WT1-specific T-cells with R-functions fisher.test and p.adjust. Differences between bulk TCRβ patient cohorts and healthy epitope-specific T-cell frequencies were assessed using a one-sided Mann-Whitney U-test, with Benjamini–Hochberg p-value correction.

## Results

### Single-cell RNA analysis of donor-enriched T-cells uncovers WT1-specific phenotypes

To assess naturally occurring endogenous WT1-specific T-cells in healthy donor PBMCs, we identified CD8 + T-cells recognizing HLA-A*02:01 restricted WT1 epitopes VLD and RMF using flow cytometric multimer analysis. Healthy donor peripheral blood (PB) samples had an average of 0.083% VLD- and 0.017% RMF-specific CD8 + T-cells (flow gating shown in Supplemental Fig. 1A). To increase the number of antigen-specific T-cells, HLA-A*02:01 positive PBMCs (healthy control n = 6) or BMMCs (AML patients n = 6) were stimulated with WT1 peptides and then re-stimulated with peptide-loaded CD14+ monocytes in the presence of cytokines (experimental schema Fig. 1A). Following the pulsation protocol, we observed increased VLD- and RMF-specific CD8 + T-cells in healthy control samples (the proportion of WT1-specific T-cells prior to pulsation, not available from AML patients) (Fig. 1B, Supplemental Fig. 1B).

Next, we sorted WT1 dextramer-positive CD8 + T-cells and performed single-cell RNA + TCRαβ sequencing to identify paired WT1-specific TCRαβ sequences and T-cell phenotypes from the peptide-pulsed samples. Following preprocessing, we obtained 7499 VLD- and 1268 RMF-specific T-cells in total, corresponding to 3069 and 809 TCRβ sequences (Supplemental Fig. 2A).

We identified phenotype-driven clusters for the WT1-specific T-cells: naïve and central memory (Tn/Tcm_1 and Tn/Tcm_2; *SELL, TCF7, LEF1, CCR7*), transitional effector memory (Transitional Tem; *GZMA, GZMB*), activated effector (Activated Teff; *CCL5, GZMA, GZMB*), activated effector memory/terminally differentiated effector memory (Activated Tem/Temra; *CCL5, GZMA, GZMB, PRF1, NKG7*), and precursor exhausted memory clusters (Tpex; *BCL2, HAVCR2, TOX*) (Fig. 1C, D). Most cells exhibited activated Tem/Temra (34%) or transitional Tem (24%) phenotypes, which also contained the most expanded clones (Fig. 1E–G).

### TCR repertoire selection drives TCRGP model training for epitope-specificity

**Fig. 2:**
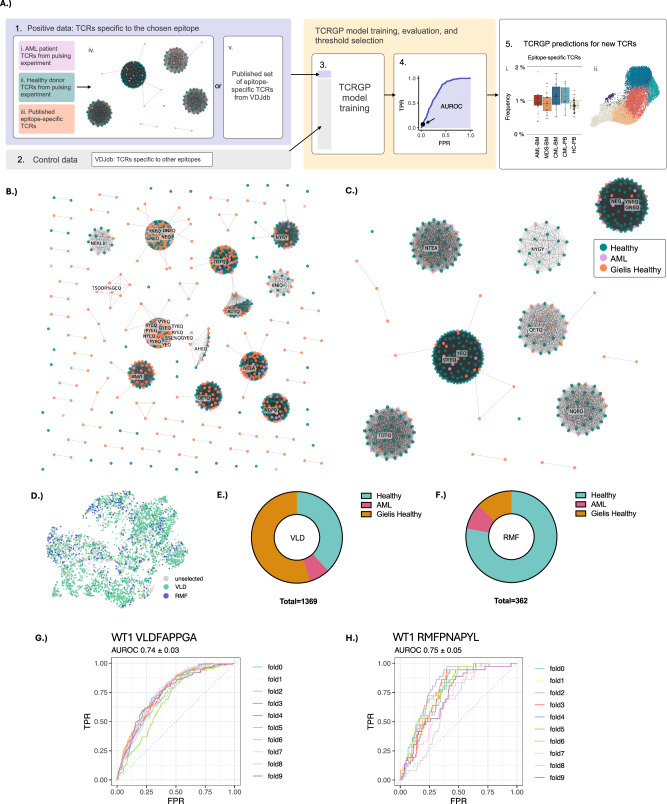
Pipeline for training novel TCRGP models. **A** Overview of the TCRGP model training process. (1) Positive data selection: TCRs specific to VLD and RMF were obtained from i) AML patients and ii) healthy donors, as well as iii) Gielis et al. [24] healthy donors were utilized. iv) GLIPH2 was used for selecting a representative TCR repertoire combining TCRs from all three data sources. v) For common viral and melanoma epitopes, epitope-specific TCRs from VDJdb (confidence score ≥ 1) were used without further filtering. (2) Control data selection: TCRs not expected to recognize the epitope of interest were selected as controls. (3) Model training: A 1:10 ratio of positive to control data was used to train a new TCRGP model for predicting TCR-specificity to the epitope of interest. (4) Model evaluation: The model was validated using stratified 10-fold cross-validation, selecting a prediction threshold corresponding to false positive rate (FPR) 0.01. (5) Model application: The trained TCRGP model was used to predict new epitope-specific TCRs. This enables analysis of i) the predicted epitope-specific TCR frequencies in different patient cohorts and ii) the phenotypes of predicted epitope-specific T-cells from scRNA+TCRαβ-seq data. **B**, **C** TCR clusters created by GLIPH2 for (**B**) VLD- and (**C**) RMF-specific TCRs. Cluster-specific motifs are annotated for clusters with ≥ 5 TCRs. Each dot represents a unique TCR clone that is colored based on the dataset it is obtained from as shown in the legend. The lines connecting the dots indicate which TCRs were clustered together. **D** UMAP representation of VLD- and RMF-specific T-cells selected by GLIPH2. **E**, **F** Ratios of GLIPH2-selected (**E**) VLD- and (**F**) RMF-specific TCRs originating from healthy donors, AML patients, and Gielis-healthy TCR sequences. **G**, **H** Evaluation of TCRGP models for (**G**) VLD- and (**H**) RMF-specificity using area under receiver operating characteristic curve (AUROC) with 10-fold stratified cross-validation on TCRGP input TCRs. Mean AUROC scores are shown above the plots, TPR true positive rate.

To identify WT1-specific TCRs in other patient cohorts, we utilized in vitro enriched epitope-specific TCRs (Supplemental Tables 1–4) to train TCRGP machine-learning models for predicting epitope-specificity in silico (Fig. 2A). As dextramer sorting can potentially capture auto-fluorescent cells with non-specific TCRs, we used GLIPH2 to group TCRs from our novel dataset and previously published WT1-specific TCRs (Gielis-healthy) [24]. GLIPH2 clusters TCRs by analyzing local CDR3β motifs, using 3-5mers and discontinuous patterns, and global similarity by computing hamming distance for sequences of the same length that share Vβ. Sequences that share similarities enriched in the query set compared to a reference data based on Fisher’s exact test are clustering together. Reassuringly, WT1-specific TCRs from our AML patients, healthy donors, and Gielis-healthy clustered together **(**Fig. 2B, C**)**, highlighting that shared TCR similarities occur despite experimental differences. The TCRs selected from the pulsing experiments are shown in Fig. 2D, the proportions of selected TCRs from different origins in Fig. 2E, F, and the selected TCR sequences in Supplemental Tables 5, 6.

Our TCRGP models were trained using epitope-specific and control TCRβs in a 1:10 ratio to factor in non-specific TCR variety and number. Evaluation with stratified 10-fold cross-validation resulted in mean AUROC values of 0.74 and 0.75 for VLD and RMF epitopes, with AUPR values of 0.2 and 0.18 respectively, (Fig. 2G, H and Supplementary Fig. 3). In addition to WT1 epitopes, TCRGP models were trained against CMV, IAV, EBV, SARS-CoV-2 and melanoma-related epitopes and achieved mean AUROC and AUPR values of 0.82 ± 0.08 and 0.58 ± 0.18, respectively (Supplementary Fig. 3). The AUROC and AUPR values provide measures of a model’s ability to discriminate between epitope-specific and non-specific TCRβs across various threshold settings.

### Clinical cohort modeling reveals variations in epitope-specific TCR frequency

To assess WT1-specificity in hematological cancers via TCRGP models, we compiled TCRβ cohorts of healthy donors, AML, MDS, and CML patients and single-cell RNA + TCRαβ cohorts of AML and CML patients (Table 1), as described in Materials and Methods.

**Fig. 3:**
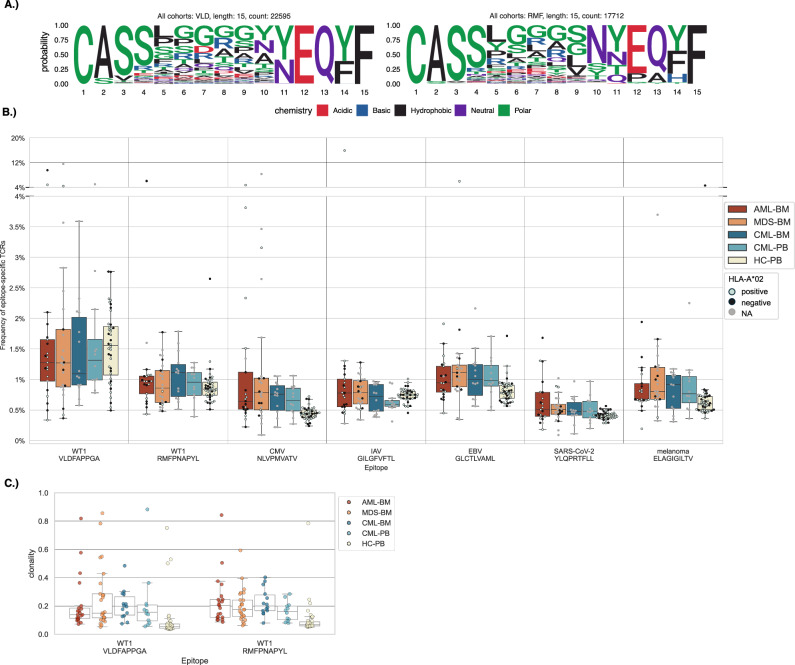
Frequency and clonality of epitope-specific TCRs in AML, MDS and CML bulk TCRβ cohorts. **A** Sequence logos (Seqlogos) of all T-cells in the bulk TCRβ cohorts with a CDR3β of length 15, predicted to be specific to VLD or RMF. **B** Boxplots showing the distribution of T-cells predicted to be epitope-specific across bulk TCRβ cohorts, epitopes indicated along the x-axis. The analyzed epitopes include WT1 VLD and RMF, cytomegalovirus (CMV), influenza A virus (IAV), Epstein-Barr virus (EBV), severe acute respiratory syndrome coronavirus 2 (SARS-CoV-2) and a melanoma-associated antigen (melanoma). Each dot represents an individual patient and is colored based on their HLA-A*02 status. **C** Boxplot of mean Simpson clonality scores for VLD- and RMF-specific T-cells across bulk TCRβ cohorts. Higher scores indicate greater clonal dominance within the predicted epitope-specific T-cell populations. Each dot represents an individual patient. Boxes indicate the quartiles of the data and whiskers the rest of the distribution, excluding points determined as outliers.

TCRGP models identified thousands of WT1-specific T-cells in each bulk TCRβ patient cohort (Supplementary Tables 7–11, Supplemental Fig. 4A–E). Only 7-9% of CDR3β sequences predicted to recognize either epitope were shared within each cohort (Supplementary Fig. 5A–C), with little overlap between the cohorts and the WT1-specific T-cells used for training the models, highlighting the importance of predictive tools to capture epitope-specific T-cells from cohort data. To visualize WT1-specific TCR similarities, we created sequence logos of bulk TCRβ cohort T-cells analyzing TCRs presenting the most abundant CDR3β length (Fig. 3A, Supplementary Fig. 6A). Both VLD- and RMF-specific TCRs exhibited motif similarity and CDR3β mid-sequence diversity (Supplementary Fig. 5A, B), with the highest sequence diversity occurring between positions 5 to 10 (Fig. 3A).

Next, we compared the frequency of WT1-specific T-cells in the different cohorts. There was no significant difference in the VLD- or RMF-specific T-cell frequencies within the patient cohorts compared to healthy, although VLD- and RMF-specific T-cells were more clonal in the patient cohorts (Fig. 3B, C, Supplementary Table 14, Supplementary Fig. 6B). Nonetheless, there was significant intra-cohort variation in the frequency and size of clones (Supplementary Fig. 6C).

#### WT1-specific TCR frequencies and phenotypes revealed in single-cell clinical cohorts

**Fig. 4:**
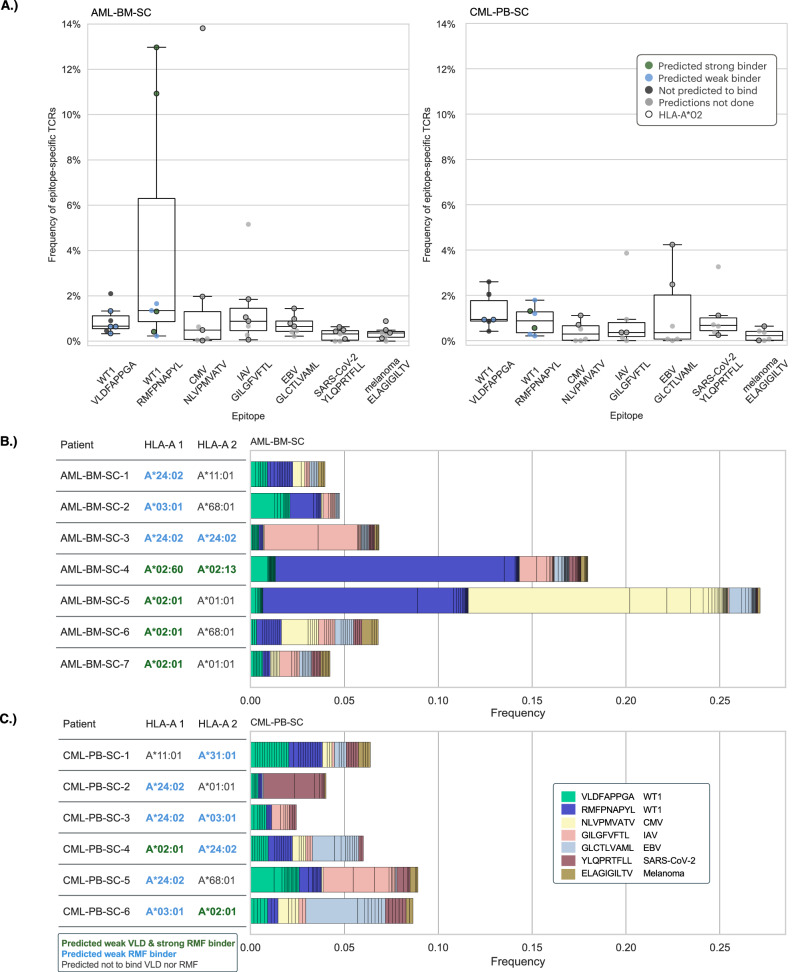
Frequency of epitope-specific TCRs in AML and CML single-cell TCRαβ cohorts. **A** Boxplots display the frequencies of T-cells predicted to be specific to the epitopes on the x-axis within the single-cell AML (left panel) and CML (right panel) cohorts. Each dot represents one patient and is colored by predicted HLA-A binding and circled if the patient has HLA-A*02 (or HLA-A*02:01 in case of AML-BM) as shown in the legend. Boxes indicate the quartiles of the data and whiskers the rest of the distribution, excluding points determined as outliers. **B**, **C** Frequency of epitope-specific TCRs predicted with TCRGP for each patient within the single-cell TCRαβ (**B**) AML and (**C**) CML cohorts. Each row is a separate patient. TCRs are colored based on their specificity as shown in the legend. Clones are separated by vertical lines. The HLA-A alleles of each patient are colored by predicted HLA-A binding as shown in the legend below.

After establishing the utility of our model in TCRβ sequencing data, we analyzed scRNA+TCRαβ-seq data from newly-diagnosed/refractory AML patients and CML patients in remission [31, 37]. Epitope frequencies are displayed in Fig. 4A–C with clinical data, HLA-I alleles, and epitope-binding capacities in Supplemental Tables 12, 13. We first predicted antigen-specific TCRs using the generated TCRGP models and compared their frequencies. Similar to the bulk TCRβ cohorts, expanded TCR repertoires were identified for both WT1-epitopes. Three AML patients (AML-BM-SC-2, AML-BM-SC-4 and AML-BM-SC-5) had markedly expanded anti-WT1 responses in their BM samples (Fig. 4B). Notably, RMF-reactive clonotypes were as high as 11-13% frequency of the total CD8 + BM TCR repertoire in two AML patients. Assuringly, we found only small reactivity against an irrelevant melanoma-associated epitope. We also detected notable anti-viral reactive clonotypes in AML BM, with >3% of anti-viral clones in four individuals. In general, we identified only small antigen-specific clones in the PB of the CML patients (Fig. 4C).

For most of the AML patients (5 out of 7 analyzed patients), only few WT1-specific CD8 + T-cells were discovered in their BM samples; therefore, we investigated the implications of patients’ HLA-A allele on WT1 peptide presentation. We inferred patient HLA-I types with Optitype and additionally predicted WT1 epitope binding affinities for each HLA allele in both single-cell cohorts (Supplemental Tables 12,13, Supplemental Table 15, Supplemental Fig. 7). Patients AML-BM-SC-4, AML-BM-SC-5, AML-BM-SC-6, and AML-BM-SC-7 expressed alleles from HLA-A*02 groups, and as such were expected to present both WT1 epitopes as natural ligands (Fig. 4B). Patients AML-BM-SC-1, AML-BM-SC-2, and AML-BM-SC-3 expressed HLA class I alleles which were predicted to bind weakly to RMF epitope (Supplemental Table 15, Supplemental Fig. 7). Thus, the absence of strong WT1-specific immunity in the majority of these patients could not solely be attributed to HLA-genotype. We further investigated *WT1* expression in the patients’ BM cells from the scRNAseq data and observed that absence of anti-WT1 responses could not be explained by the lack of *WT1* expression in AML BM cells (Supplemental Fig. 8). In the CML cohort, only CML-PB-SC-4 and CML-PB-SC-6 expressed HLA-A*02:01, but all patients were predicted to have HLA-A alleles capable of presenting RMF.

#### WT1-specific T-cells in AML bone marrow samples display effector phenotypes

**Fig. 5:**
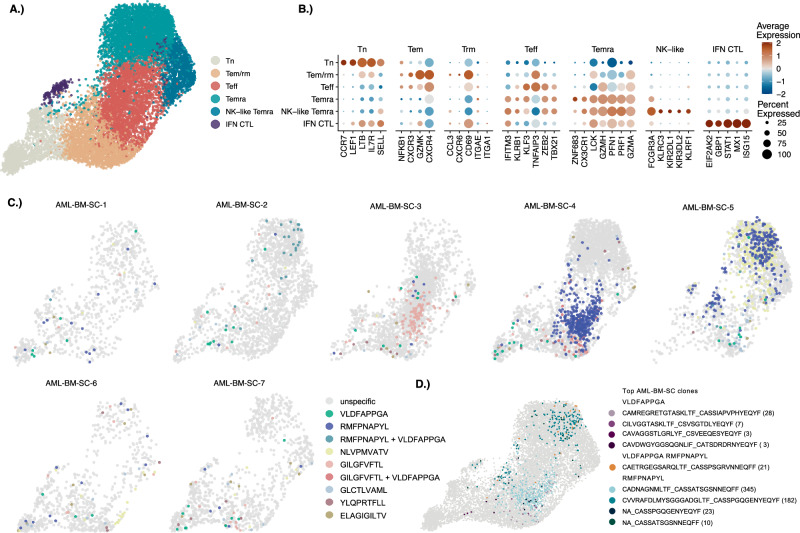
Phenotypes and epitope-specificity of CD8 + T-cells in the single-cell AML cohort. **A** UMAP representation of the CD8 + T-cell phenotypes within the single-cell AML cohort, colored by the manually annotated clusters: naïve/central memory (Tn), effector memory resident memory (Tem/rm), effector (Teff), terminally differentiated effector memory (Temra), Natural Killer-like terminally differentiated effector memory (NK-like Temra), and Interferon cytotoxic T lymphocytes (IFN CTL). **B** Plot shows the scaled average expression and proportion of the cells within each cluster expressing the cluster-defining markers. **C** UMAP representation of the CD8 + T-cells predicted to be specific to the epitopes shown in the legend for individual patients within AML BM single-cell cohort. **D** UMAP displaying the four most abundant (Top clones) predicted VLD- and RMF-specific clonotypes along with one shared VLD-RMF-specific clonotype. Legend uses CDR3α_CDR3β format, with an NA for those missing an α-chain and number of occurrences in parentheses.

To detail the phenotypic features of epitope-specific CD8 + T-cells in AML BM we analyzed patient scRNA+TCRαβ-seq data resolving cell clusters: naïve/central memory (*SELL, LEF1, CCR7*), effector/resident memory (*CD69, CXCR6*), effector (*KLF3*), TEMRA (*GZMA, GZMH, PRF1, NKG7*), NK-like TEMRA (*FCGR3A, KIR2DL1/2* among cytolytic molecules) and interferon clusters (*ISG15, MX1*) (Fig. 5A, B). We then integrated each TCRGP classifier prediction to its corresponding CD8 + T-cell in the scRNA-seq data to visualize cellular phenotype and specificity. In general, expanded WT1-specific CD8 + T-cells were enriched to effector or effector/resident memory clusters **(**RMF-specific Teff *p* = 2.09E-31 and VLD-specific Tem/rm *p* = 6.77E-3 Fisher’s exact test with Benjamini–Hochberg correction; Supplemental Table 16) and were preferentially RMF-specific **(**Fig. 5C**)**. The largest RMF-specific clones were restricted to one or two phenotypic states in the patients’ BM **(**Fig. 5D**)**. Although VLD-specific T-cells were rather equally distributed among various clusters, presenting as ~1% of all CD8 + T-cells (Supplemental Table 12), these were most enriched to the Tem/rm cluster (*p* = 6.77E-03 Fisher’s exact test with Benjamini-Hochberg correction, Supplemental Table 16). The expanded virus-specific T-cells (targeting CMV, IAV, EBV, and SARS-CoV-2 epitopes) exhibited similar phenotypes as WT1-specific T-cells. However, in the differential gene expression analysis, the anti-WT1 T-cells exhibited higher expression of *ZNF683* and *S1PR* (Supplemental Fig. 9), both of which have been associated with effective antitumor responses and persistence in tumor-infiltrating lymphocytes [38–40].

To validate our results, particularly for the largest RMF-specific clones, we compared the current TCRGP predictions based on TCRβ to predictions obtained using TCRαβ models described in the Supplemental material. The RMF-specificity predictions showed a high degree of correlation, and the three largest clones with paired TCRαβ (Fig. 5D) received high prediction scores from both models. However, for the largest clone, the TCRαβ model’s prediction was just below the predictive threshold (Supplemental Figs. 10, 11).

#### WT1-specific T-cells are retained in low numbers in memory phenotype in CML patients under remission

**Fig. 6:**
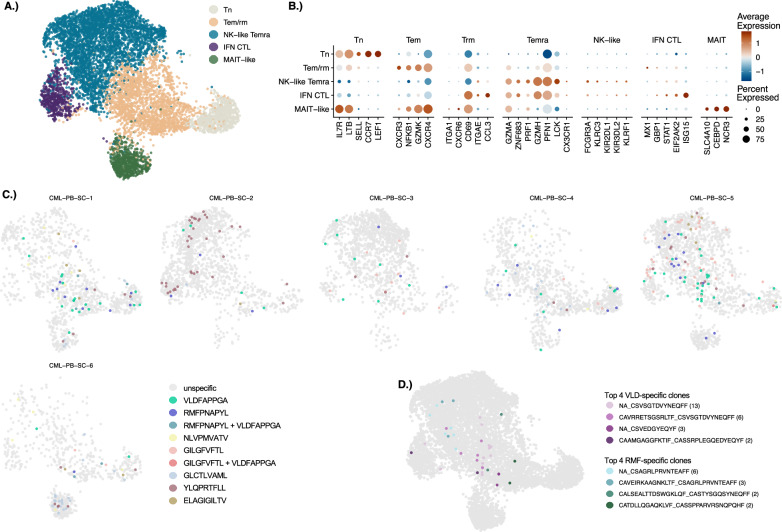
Phenotypes and epitope-specificity of CD8 + T-cells in the single-cell CML cohort. **A** UMAP representation of the CD8- T-cell phenotypes within the single-cell CML cohort, colored by the manually annotated clusters: naïve/central memory (Tn), effector memory resident memory (Tem/rm), NK-like terminally differentiated effector memory (NK-like Temra), Interferon cytotoxic T-lymphocytes (IFN CTL), and Mucosal-associated invariant like CD8 + T-lymphocytes (MAIT-like). **B** Plot shows the scaled average expression and proportion of the cells within each cluster expressing the cluster defining markers. **C** UMAP representation of the CD8 + T-cells predicted to be specific to the epitopes shown in the legend for individual patients within CML PB single-cell cohort. **D** UMAP displaying the four most abundant (Top clones) predicted VLD- and RMF-specific TCR clonotypes. Legend uses CDR3α_CDR3β format, with an NA for those missing an α-chain and number of occurrences in parentheses.

To detail the phenotypic features of epitope-specific CD8 + T-cells within CML patients in remission under TKI treatment, we analyzed patient scRNA+TCRαβ-seq data, resolving the cell clusters: NK-like TEMRA (*FCGR3A, KIR2DL1/2*, among cytolytic molecules *GZMA, GZMH, PRF1*) and effector/resident memory (*CD69, CXCR6*) T-cells as the most frequent phenotypes (Fig. 6A, B). The patients also presented naïve/central memory, interferon-responsive and MAIT-like CD8 + T-cells. WT1-specific cells were present at low frequencies, as expected for patients in remission. WT1-specific CD8 + T-cells were present in all, but two patients (CML-PB-SC-1 and CML-PB-SC-5) showed higher frequencies, 3.8% total CD8 + T-cells, than the rest **(**Fig. 6C, D, Supplemental Table 13). WT1-specific T-cells were most enriched in Tem/rm (VLD-specific *p* = 5.73E−06 and RMF-specific *p* = 1.98E−1, Fisher’s exact test with Benjamini–Hochberg correction) but also within Tn (VLD-specific *p* = 8.48E−02 and RMF-specific *p* = 3.43E−02,) (Fig. 6A-D, Supplemental Table 16).

## Discussion

The graft-versus-leukemia effect in HSCT provides strong evidence for the immune system’s capacity to eradicate leukemia cells [41]. Recently, targeted anti-leukemic immunotherapies have been developed to alleviate HSCT-related toxicity while retaining positive therapeutic outcomes. Some of these rely on cytotoxic CD8 + T-cells targeting of leukemia-associated antigens displayed on the leukemia cell surface. WT1 presents several characteristics of an ideal target antigen for immunotherapies [9, 10, 42, 43]; it is highly expressed in leukemia cells, compared to normal cells, and leukemia cells rely on its continuous expression [44–46] making tumor escape through antigen loss unlikely. Furthermore, WT1 presents several epitopes that are effectively recognized by the immune system, producing durable responses. As a result, multiple clinical trials exploiting WT1 have been conducted in leukemia [11, 12, 47–49]. Despite the high clinical relevance of WT1, endogenous T-cell responses against WT1 have not been analyzed at scale. Experimental methods, such as tetramer-staining coupled with flow cytometric analysis, are laborious and require large amounts of patient material. Although bulk TCRβ and scRNA+TCRαβ sequencing produce TCR repertoire information, inferring epitope-specificity remains challenging due to the lack of publicly available leukemia-antigen-targeting TCR data and the cross-reactive nature of TCRs. Recently, in silico methods utilizing, e.g., Gaussian process-based methods or random forests have been developed to predict whether an ‘orphan’ TCR recognizes a specified epitope [20, 24, 31, 50]. These methods have already been applied to analyze antigen-specific TCR repertoires in patients with hematological malignancies and solid tumors [24, 31, 50].

To analyze endogenous T-cell responses against WT1 on a large scale, we trained Gaussian process-based computational TCRGP models to predict WT1-specific TCR repertoires from TCR sequencing data. As only a handful of WT1-specific TCRs were publicly available in the VDJ database [18], we first needed to generate an epitope-specific CD8 + TCR dataset. We enriched WT1-specific CD8 + T-cells from healthy donors and AML patients using peptide stimulation, WT1 dextramer-staining, and FACS. We observed that in vitro T-cell stimulation enriched epitope-specific responses to both VLD and RMF epitopes, yet VLD-specific T-cells were more frequent, a result noted previously [4, 24]. To mitigate the potential unbalance of the epitope-specific TCR sequence diversity for different WT1 epitopes, we additionally incorporated publicly available TCR repertoire data [24] for the model training of both epitopes.

In this study, we utilized both bulk TCRβ and scRNA+TCRαβ sequenced datasets of AML, CML, and MDS patients to investigate their WT1-specific T-cell frequencies and phenotypic features. Although no significant differences in WT1-specific T-cell frequencies were identified between patient cohorts and healthy individuals when analyzing TCRβ sequencing data, VLD- and RMF-specific T-cells exhibited greater clonality in the patient cohorts. This may indicate that diverse T-cell populations within healthy individuals capable of recognizing WT1 are rarely as expanded as those of patients. Notably, in some AML patients, WT1-specific T-cells accounted for up to 13% of the total CD8 + T-cell BM repertoire. This finding was confirmed by scRNA+TCRαβ-seq data, which also revealed expanded RMF-specific T-cell clones in AML patient BM samples. When strong WT1 CD8 + T-cell responses were present, they were dominated by a few expanded TCR clonotypes, with the largest WT1-specific clones restricted to effector or memory phenotypic states. This suggests that anti-leukemia responses occurred naturally in some individuals, yet the response was not strong enough to control malignant progression. The low frequency of WT1-specific T-cells observed in some individuals may relate to the self-antigen nature of WT1, thymic selection of high-affinity TCRs to self-antigen, or TCR repertoire contraction during T-cell differentiation. Additionally, we lacked comprehensive HLA-I genotyping for each cohort, potentially skewing the interpretation for some individuals. The observed interindividual variability in WT1-specific T-cell frequency underscores the personalized nature of immune responses, where genetic, epigenetic, and external factors contribute to differential epitope recognition.

WT1-specific CD8 + T-cells exhibited diverse phenotypes, including naïve, effector, and memory. However, following pulsation protocol, the most expanded WT1-specific clones predominantly displayed an effector or terminal effector memory phenotype. In melanoma, the adoptive transfer of central memory T-cells leads to longer persistence and higher proliferative capacity, showing superior anti-tumor capacity compared to effector memory cells [51, 52]. While this suggests pulsation protocols may not be ideal for therapeutic cell selection, ours proved highly effective in generating epitope-specific TCR sequences, which could be valuable to future immunotherapeutic strategies.

Our study has limitations as the current findings are constrained by the HLA-A:02*01 genotype. We additionally chose to generate the classifier for two common HLA-A:02*01 restricted WT1 epitopes, although leukemia cells may present additional epitopes [49, 53], thus we potentially missed naturally occurring immune responses against these additional epitopes. We also used GLIPH2 to select TCR sequences with the highest similarity for TCRGP classifier development, potentially underestimating the heterogeneity of TCR-WT1 binding in our predictions. Furthermore, we set a stringent threshold for epitope-specificity prediction to reduce false positive predictions, which also reduced the recovery of epitope-specific T-cells. Based on a comparison of TCRGP models using TCRβ or TCRαβ, the RMF models demonstrated greater robustness. RMF was also predicted to bind to a variety of HLA I types, allowing RMF-specificity prediction beyond individuals possessing HLA-A*02:01 genotype, thus making it an optimal epitope for studying anti-WT1 responses within TCR repertoires.

In summary, our innovative computational model enables the identification of WT1-specific T-cells from clinical TCR sequencing datasets and facilitates the large-scale monitoring of leukemia-antigen-specific immune responses.

## Supplementary information

Supplementary material

Supplementary tables

Supplementary table 17

## Data Availability

The processed scRNA+TCRαβ-seq data from WT1-specific T-cells used to generate prediction models and the AML-BM-SC cohort is available at Zenodo (10.5281/zenodo.15074880). All other data and sequences to evaluate conclusions are presented in the paper, Supplementary Materials, or available from publications listed in Table 1. Codes and the trained TCRGP models are available at https://github.com/emmijokinen/wt1_manu.
