# Supplementary material for: Predicting antigen-specific T-cell immunity against Wilms tumor 1 in hematologic cancer
Source: Leukemia. 2025 Aug 22;39(11):2767–78. doi: 10.1038/s41375-025-02727-y (PMC12589112; doi:10.1038/s41375-025-02727-y)
Supplement: Supplementary file 1 — Supplementary material [file 41375_2025_2727_MOESM1_ESM.pdf]

Supplementary materials:

Predicting antigen-specific T-cell immunity against Wilms tumor 1 in hematologic cancer

Supplementary Materials and Methods on pages 2-4

Supplemental Figures 1-11 on pages 5-17

Supplemental Tables 1-13 Legends on page 18-19

Supplemental Tables 14-16 on pages 20-22

Supplemental Table 17 Legend on page 23

Supplemental Materials and Methods:

Donor Acquisition HLA-matched buffy coats, homozygous for HLA-A*02:01, were obtained from the Blood Service Biobank of the Finnish Red Cross Blood Service, Vantaa, Finland. Donations by healthy donors occurred in the morning, followed by Ficoll-paque centrifugation (GE Heathcare, Chicago, Illinois, USA) in the afternoon. The isolated peripheral blood mononuclear cells (PBMC) were either frozen and stored at -150°C or immediately used in the antigen pulsing procedure. All PBMCs were cultured in RPMI 1640 supplemented with 2mM L-glutamine, 10% FBS, and 1mM sodium pyruvate (Gibco, Paisley, Scotland), henceforth referred to as R10.

Bone marrow mononuclear cells from untreated AML patients at diagnosis with HLA-A*02:01 were used in the pulsation experiments. The pre-isolated and frozen BMMCs were cultured in R10.

This study complied with the Declaration of Helsinki and the HUS ethics committee (DNRO 303/13/03/01/2011).

Antigen pulsing Adapted from Schmied et al. (1):

Donor PBMCs or patient BMMCs were stimulated in R10 supplemented with 0.5ug/mL αCD28 Ab and 3ug/mL β2M, and 6uM VLDFAPPGA (VLD) or 6uM RMFPNAPYL (RMF) peptide (JPT, Berlin, Germany) for 24 - 36 hrs. Note peptide were used separately, not as a pool. Anti-CD8 beads (cat. 130-045-201 Miltenyi Biotech, Bergisch Gladbach, Germany) were used to isolate CD8+ T-cells and cultured in 25 ng/mL IL-21 supplemented media for 48 hrs. Next 10 ng/mL IL-15 was added to the CD8+ T-cell cultures for 48 hrs. Then these cells were split and incubated in IL-15-supplemented R10 media for 48 hrs.

Monocytes from the corresponding frozen healthy PBMC or AML patient BBMC were isolated by anti-CD14 beads (cat. 130-050-201Miltenyi Biotech) and cultured in 0.5ug/mL αCD28 Ab and 3ug/mL β2M supplemented R10 media, then stimulated with 10uM VLD or RMF peptide for 3-4 hrs. Next 1M CD8+ T-cells from the previous peptide stimulation in culture were added to the loaded CD14+ cells and co-cultured for 48 hrs. After which these cells were split and incubated in IL-15-supplemented R10 media for 48 hrs.

Note all cell culture media and supplements were purchased from Gibco, Paisley, Scotland. Interleukins were sourced from PeproTech, Cranbury, NJ, USA. The following peptides were purchased from JPT, Berlin, Germany: Antigen Peptide WT1 - HLA-A*02:01 (VLDFAPPGA), Antigen Peptide WT-1 H-2 Db HLA-A*0201 HLA-B*2705 (RMFPNAPYL). All peptide stimulations were as VLD-only or RMF-only, not as a pool.

Fluorescence activated cell sorting. As preparation for FACS, live cells were isolated via Dead Cell Removal kit (cat.130-090-101 Miltenyi Biotech). Live cells were then diluted and stained with VLDFAPPGA or RMFPNAPYL Dextramer mixture per manufacturer (Immudex, Copenhagen, Denmark) instruction. Dextramer mixtures utilized: WT1 HLA-A*0201 VLDFAPPGA, cat. WB03469; WT1 HLA-A*0201 RMFPNAPYL, cat. WB2177; Negative Control Dextramer, HLA-A02*01, ALIAPVHAV cat. WB02666. Next antibodies for gating CD8+ T-cells from PBMCs or BMMCs were incubated 20 mins at + 20°C under dark conditions. These antibodies include 1:50 CD14+, BD cat. 562698; 1:10 CD3+, BD cat. 332771; 1:50 CD4+, BD cat. 562970; 1:50 CD8+, BD cat. 641400. Lastly Dextramer-positive cells were using BD Influx. VLD and RMF positive cell gates are shown in Supplementary Figure 1.

scRNA+TCRαβ-seq processing of AML-BM-SC and CML-PB-SC CD8 T cells. The R package Seurat (v5.1) (ref. 2) was used for scRNA+TCRαβ-seq data processing. For the AML-BM-SC the processing was started directly from CD8 T cells and cells from the patient samples described in Supplemental Table 12. For the CML-PB-SC we selected CD8 T cells from the samples collected at the time of TKI discontinuation (3), described in Supplemental Table 13. CD8 T cells were identified based on the published annotations, we included cells annotated as CD8 Tem/emra, CD8 Tem/rm, CD8 Tcm/n, Unconv: NKT, Unconv: MAIT, and Unconv: Tgd and in addition required the cells to have expression of *CD3E*, *CD8A*, or *CD8B* > 1. For both datasets the data was log-normalized and 2000 most highly variable genes not including TCR V-, D-, and J-genes were calculated by "FindVariableFeatures", using 'vst' selection. Cell cycle phases were scored by "CellCycleScoring" and phase effects were regressed out during data scaling with "ScaleData". Harmony was used for the batch correction of the patient samples. UMAP representation was computed based on top 20 harmony components using "RunUMAP" function. Clusters were likewise defined with top 20 harmony components using "FindNeighbors" and "FindClusters" functions with resolution 0.5 for AML-BM-SC and 0.2 for CML-PB-SC. From CML-PB-SC Cluster 5 that consisted of 35 cells with mixed phenotypes was removed, leaving us with 7431 CD8 T cells. The AML-BM-SC dataset contains 12 380 CD8 T cells

WT1 expression of AML-BM-SC cohort patient cells. Azimuth (4) was used to assign cell types for all patient cells based on its bone marrow reference (5,6,7). HSPCs, Monocytes and DCs were selected based on level 1 predictions and based on the level 2 predictions additional 36 cells were removed that were predicted to be T cells (CD4 Memory, CD8 Naive, CD8 Effector_2), or late erythrocytes (Late Eryth). WT1 expression was then evaluated for the remaining 10 834 cells. Batch correction was done with Harmony to correct for each patient and UMAP representation was computed using first 20 Harmony components.

TCRGP models for WT1 epitopes with paired TCRαβ. The TCRGP models using paired TCRαβ were trained for VLD and RMF in the same manner as the TCRB models. From the GLIPH2 selected TCRs for each epitope we selected all TCRs with CDR3 and V-gene available from both TCRα and TCRβ chains. Notably this excluded completely the Gielis-Healthy data that does not contain TCRα. This resulted to 508 TCRαβ s for VLD and 244 TCRαβs for RMF. The models were trained with 1:10 ratio of positive and negative data, which was collected from VDJdb (8). Evaluation via stratified 10-fold cross-validation and comparison of the predictions made for the AML-BM-SC and CML-PB-SC cohorts using both TCRB and TCRαβ models are shown in Supplemental Figures 10-11.

Supplemental references

1. Schmied S, Gostick E, Price DA, Abken H, Assenmacher M, Richter A. Analysis of the functional WT1-specific T-cell repertoire in healthy donors reveals a discrepancy between CD4(+) and CD8(+) memory formation. Immunology. 2015 Aug;145(4):558–69.
2. Hao Y, Stuart T, Kowalski MH, Choudhary S, Hoffman P, Hartman A, et al. Dictionary learning for integrative, multimodal and scalable single-cell analysis. Nat Biotechnol. 2024 Feb;42(2):293–304
3. Huuhtanen J, Adnan-Awad S, Theodoropoulos J, Forstén S, Warfvinge R, Dufva O, et al. Single-cell analysis of immune recognition in chronic myeloid leukemia patients following tyrosine kinase inhibitor discontinuation. Leukemia. 2024 Jan;38(1):109–25.
4. Hao Y, Hao S, Andersen-Nissen E, Mauck WM, Zheng S, Butler A, Lee MJ, Wilk AJ, Darby C, Zager M, Hoffman P. Integrated analysis of multimodal single-cell data. Cell. 2021 Jun 24;184(13):3573-87.
5. Oetjen KA, Lindblad KE, Goswami M, Gui G, Dagur PK, Lai C, Dillon LW, McCoy JP, Hourigan CS. Human bone marrow assessment by single-cell RNA sequencing, mass cytometry, and flow cytometry. JCI insight. 2018 Dec 6;3(23):e124928.
6. Granja JM, Klemm S, McGinnis LM, Kathiria AS, Mezger A, Corces MR, Parks B, Gars E, Liedtke M, Zheng GX, Chang HY. Single-cell multiomic analysis identifies regulatory programs in mixed-phenotype acute leukemia. Nature biotechnology. 2019 Dec;37(12):1458-65.
7. Human Cell Atlas Immune Cell Consensus. https://explore.data.humancellatlas.org/projects/cc95ff89-2e68-4a08-a234-480eca21ce79
8. Shugay M, Bagaev DV, Zvyagin IV, Vroomans RM, Crawford JC, Dolton G, et al. VDJdb: a curated database of T-cell receptor sequences with known antigen specificity. Nucleic Acids Research. 2018 Jan 4;46(D1):D419–27.
9. Szolek A, Schubert B, Mohr C, Sturm M, Feldhahn M, Kohlbacher O. OptiType: precision HLA typing from next-generation sequencing data. Bioinformatics. 2014 Dec 1;30(23):3310–6.
10. Reynisson, B., Alvarez, B., Paul, S., Peters, B., & Nielsen, M. NetMHCpan-4.1 and NetMHCIIpan-4.0: improved predictions of MHC antigen presentation by concurrent motif deconvolution and integration of MS MHC eluted ligand data. Nucleic acids research. 2020, 48(W1), W449-W454.

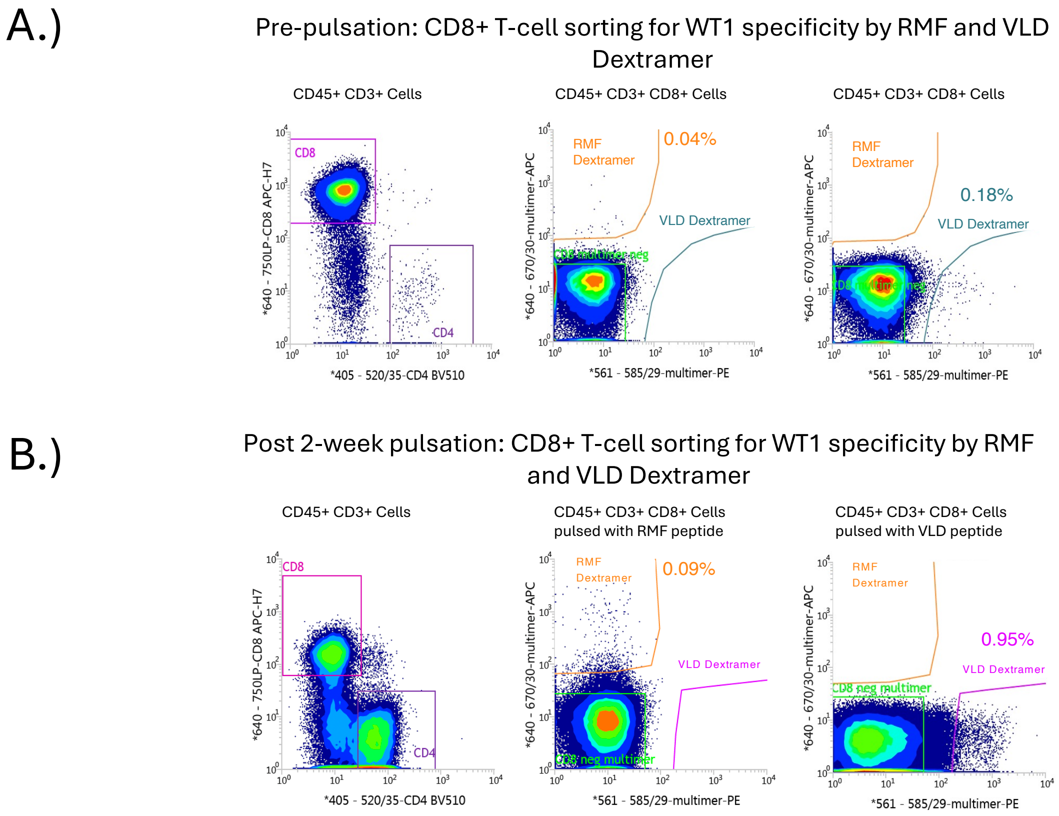

Supplemental Figure 1. WT1 multimer sorting of CD8^{+} T-cells from a healthy donor This figure presents fluorescence event plots from a randomly selected healthy donor, highlighting heterogeneity in both the basal levels of WT1 epitope-specific CD8^{+} T-cells and the extent of enrichment following peptide pulsation within the same individual to different epitopes. **A**: Fluorescence event plots of CD8^{+} T-cells stained with Immudex Dextramer for WT1 epitopes RMF (center) and VLD (right) before peptide pulsing. **B**: Fluorescence event plots of the same donor's CD8^{+} T-cells stained with Immudex Dextramer for WT1 epitopes RMF (center) and VLD (right) after the 2-week peptide pulsing procedure.

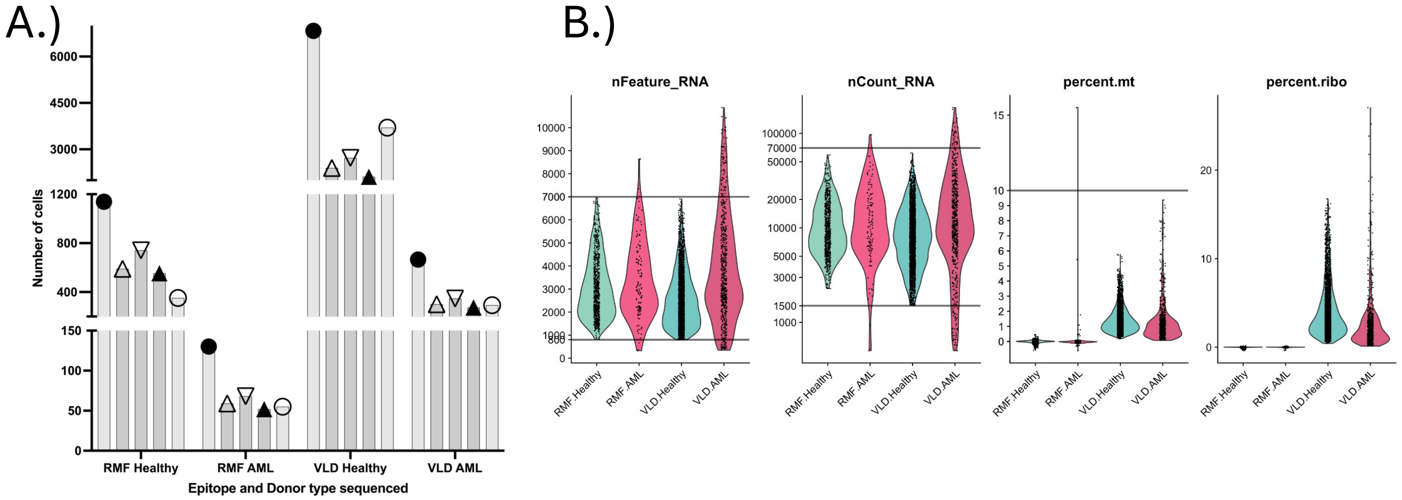

	● total cells	△ cells with TCRα	▽ cells with TCRβ	▲ cells with TCRαβ	○ cells without TCR
RMF Healthy	1138	590	741	554	351
RMF AML	130	59	68	52	55
VLD Healthy	6833	2398	2722	2114	3699
VLD AML	666	300	347	274	293

Supplemental Figure 2. WT1-specific counts and scRNA+TCRαβ-seq pre-processing A: T-cell counts of epitope-specific sequenced cells by donor type. The counts for WT1 epitopes RMF and VLD are displayed, categorized by donor type. The populations shown include total sequenced cells, cells with TCR α-chain sequences, cells with TCR β-chain sequences, cells with paired TCR αβ sequences, and cells without any detected TCR sequences. **B**: WT1-specific single-cell RNA analysis for the epitopes RMFPNAPYL (RMF) and VLDFAPPGA (VLD), separated by donor origin, either healthy or acute myeloid leukemia (AML). Quality control plots are displayed to visualize the filters applied in this study.

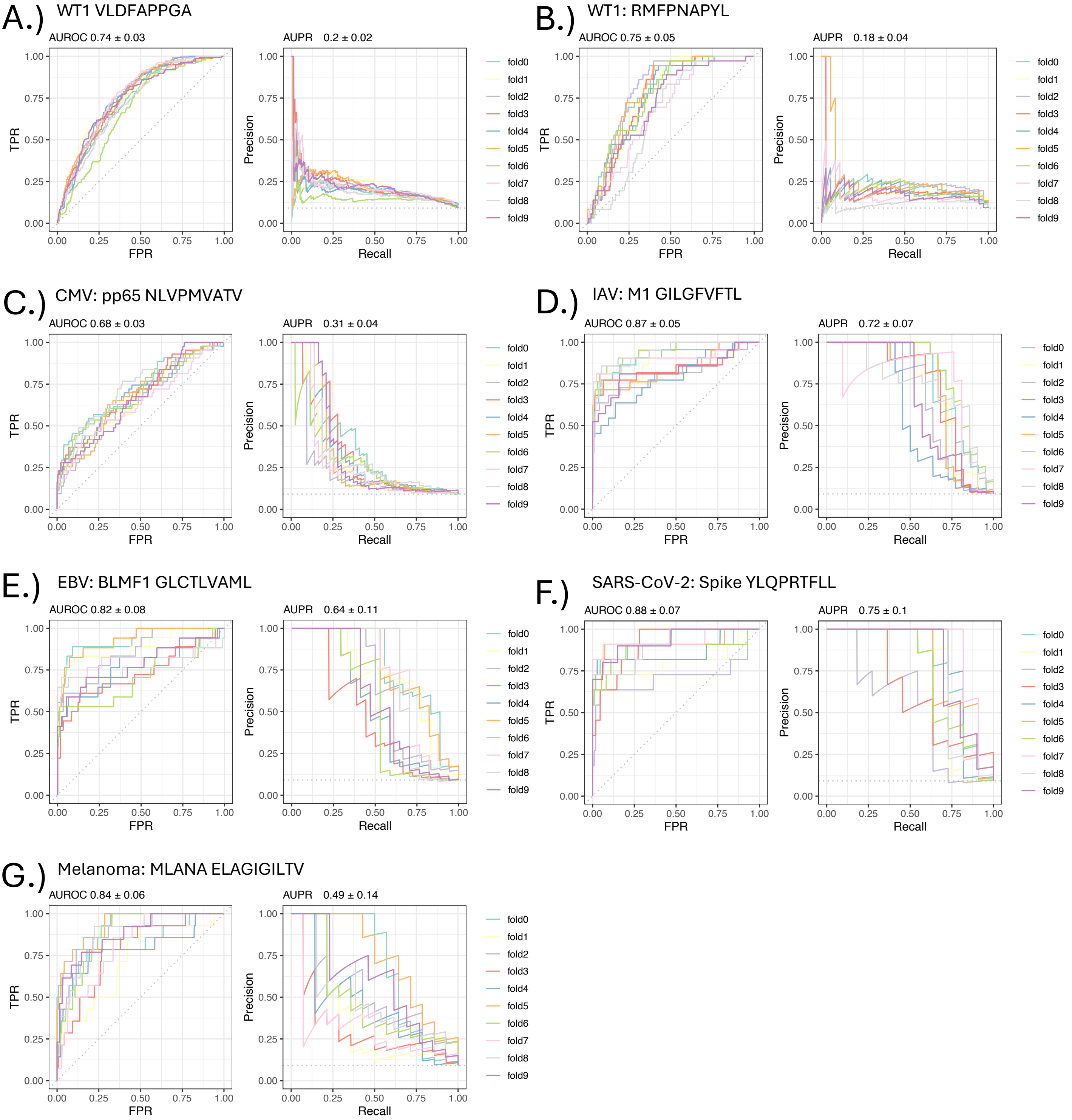

Supplemental Figure 3. Accuracy of trained TCRGP models evaluated via 10-fold cross-validation (AUROC and AUPR) Each model was trained using a dataset containing one part epitope-specific TCRs and ten parts TCRs assumed to be non-specific to the selected epitope. Stratified 10-fold cross-validation was performed to assess model performance in terms of the area under the receiver operating characteristic curve (AUROC) and the area under the precision-recall curve (AUPR). Figures **A-G** correspond to the following epitopes: **A**: VLDFAPPGA, **B**: RMFPNAPYL, **C**: NLVPMVATV, **D**: GILGFVFTL, **E**: GLCTLVAML, **F**: YLQPRTFLL, and **G**: ELAGIGILTV.

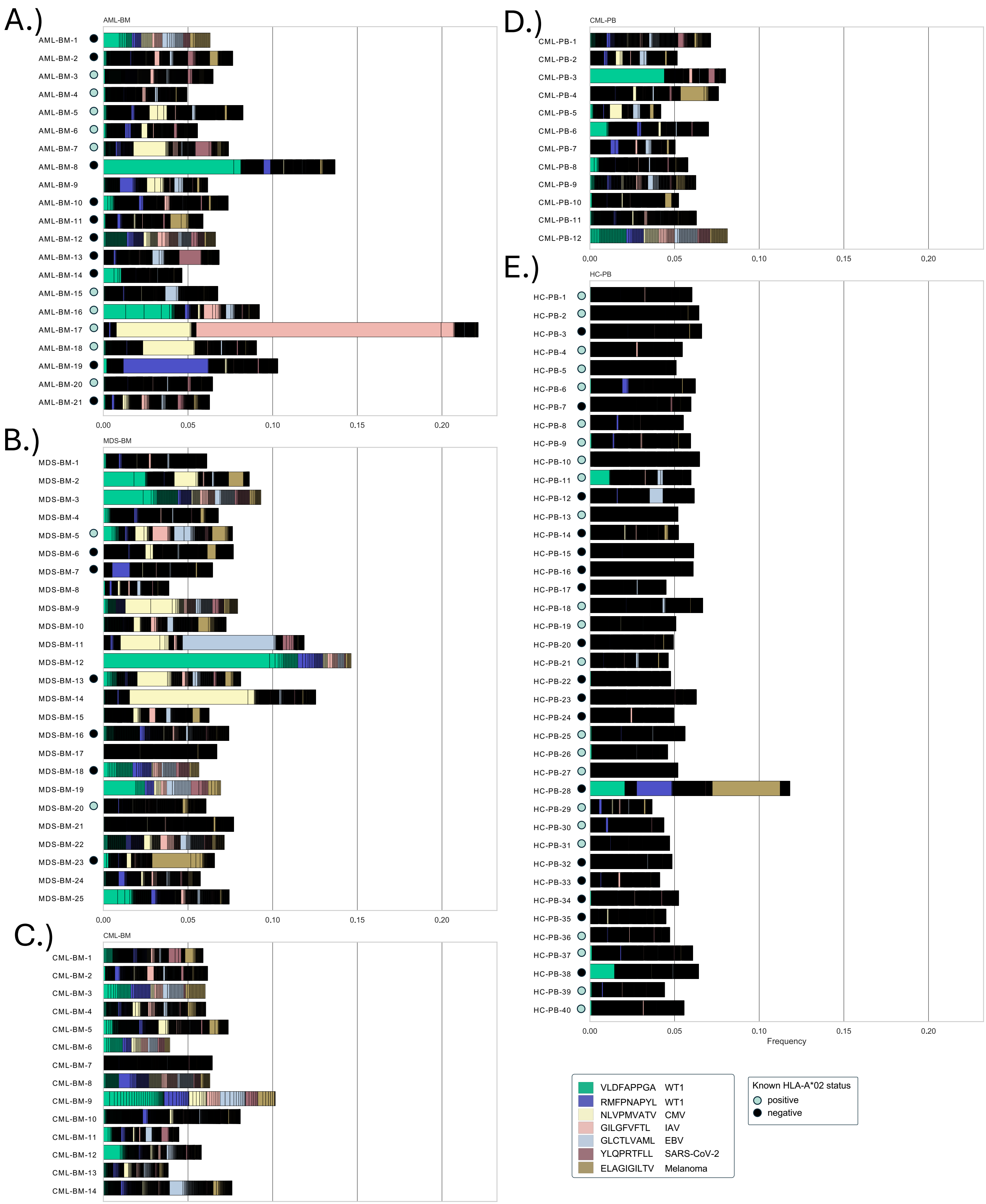

Supplemental Figure 4. Frequency of epitope-specific TCRs predicted by TCRGP per patient The predicted frequency of epitope-specific TCRs per patient is displayed by cohort in Figures **A-E**, representing **A**: AML-BM, **B**: MDS-BM, **C**: CML-BM, **D**: CML-PB, and **E**: HC-PB. TCRs are color-coded based on epitope specificity, as indicated in the legend, with clones separated by vertical lines. Patients whose HLA-A*02:01 status is known are marked with circles as shown in the legend.

A.)

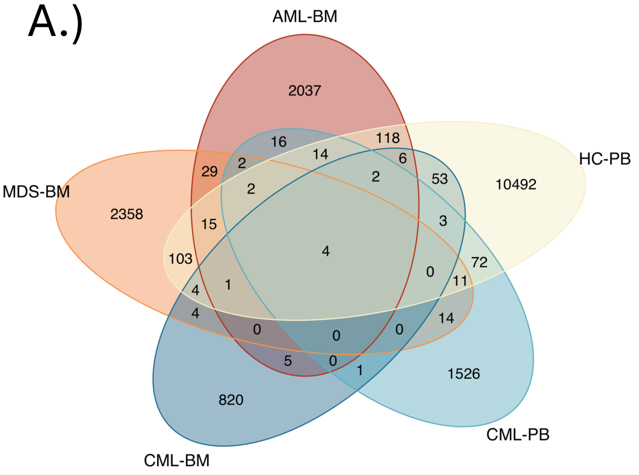

Shared CDR3Bs	AML-BM	MDS-BM	CML-BM	CML-PB	HC-PB
CASSPGLAGTYEQYF	2	3	3	1	12
CASSRTSGNNEQFF	1	1	1	1	3
CASSLGLAGNNEQFF	4	5	3	1	7
CASSLLAGGNNEQFF	1	1	11	11	2

B.)

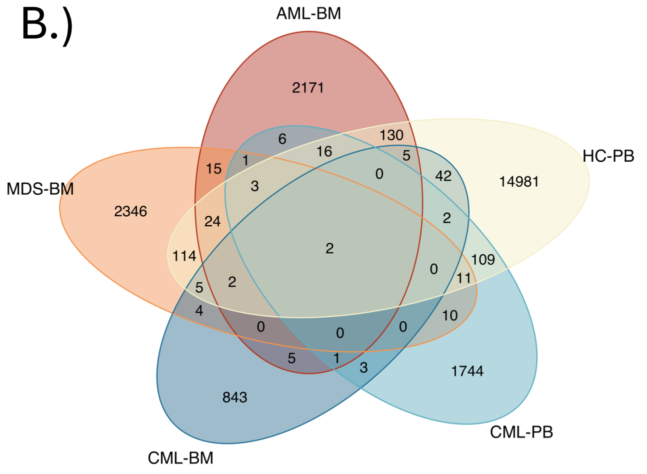

Shared CDR3Bs	AML-BM	MDS-BM	CML-BM	CML-PB	HC-PB
CASSLGQAYEQYF	2	7	2	5	3
CASSLGYEQYF	4	2	6	3	17

C.)

Cohort	Distinct RMF specific CDR3Bs	RMF specific CDR3Bs overlapping with training data (362 CDR3Bs)	Distinct VLD specific CDR3Bs	VLD specific CDR3Bs overlapping with training data (1369 CDR3Bs)	Distinct RMF and VLD specific CDR3Bs
AML-BM	2251	0	2372	9	343 (7.4%)
MDS-BM	2547	0	2523	14	358 (7.0%)
CML-BM	903	1	912	2	133 (7.3%)
CML-PB	1667	0	1902	6	265 (7.4%)
HC-PB	10899	1	15413	33	2336 (8.9%)

Supplemental Figure 5. Overlap of CDR3β sequences among predicted WT1-specific T-cells A: Overlap of CDR3β sequences from predicted RMF-specific T-cells across bulk TCRβ cohorts, visualized as a Venn diagram and a table listing shared CDR3β sequences along with their occurrences in each cohort. **B**: Equivalent visualization for VLD-specific T-cells. **C**: Total numbers of distinct RMF/VLD-specific CDR3β sequences, their overlap with the training dataset, and the number and frequency of CDR3β sequences predicted to be specific to both RMF and VLD within each cohort.

A.)

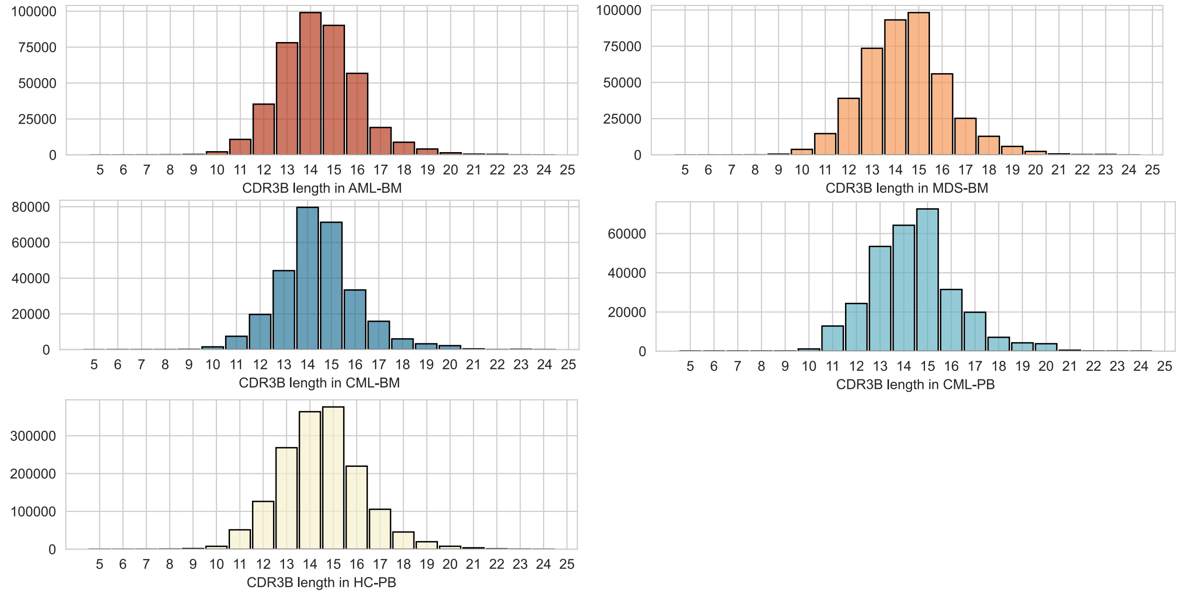

B.)

Cohort	Simpson clonality of CDR3β sequences of		
	RMF-specific T cells	VLD-specific T cells	All T cells
AML-BM	0.2353	0.2401	0.0902
MDS-BM	0.2049	0.2803	0.0915
CML-BM	0.2272	0.2334	0.0996
CML-PB	0.1614	0.2360	0.0945
HC-PB	0.1018	0.0922	0.0186

C.)

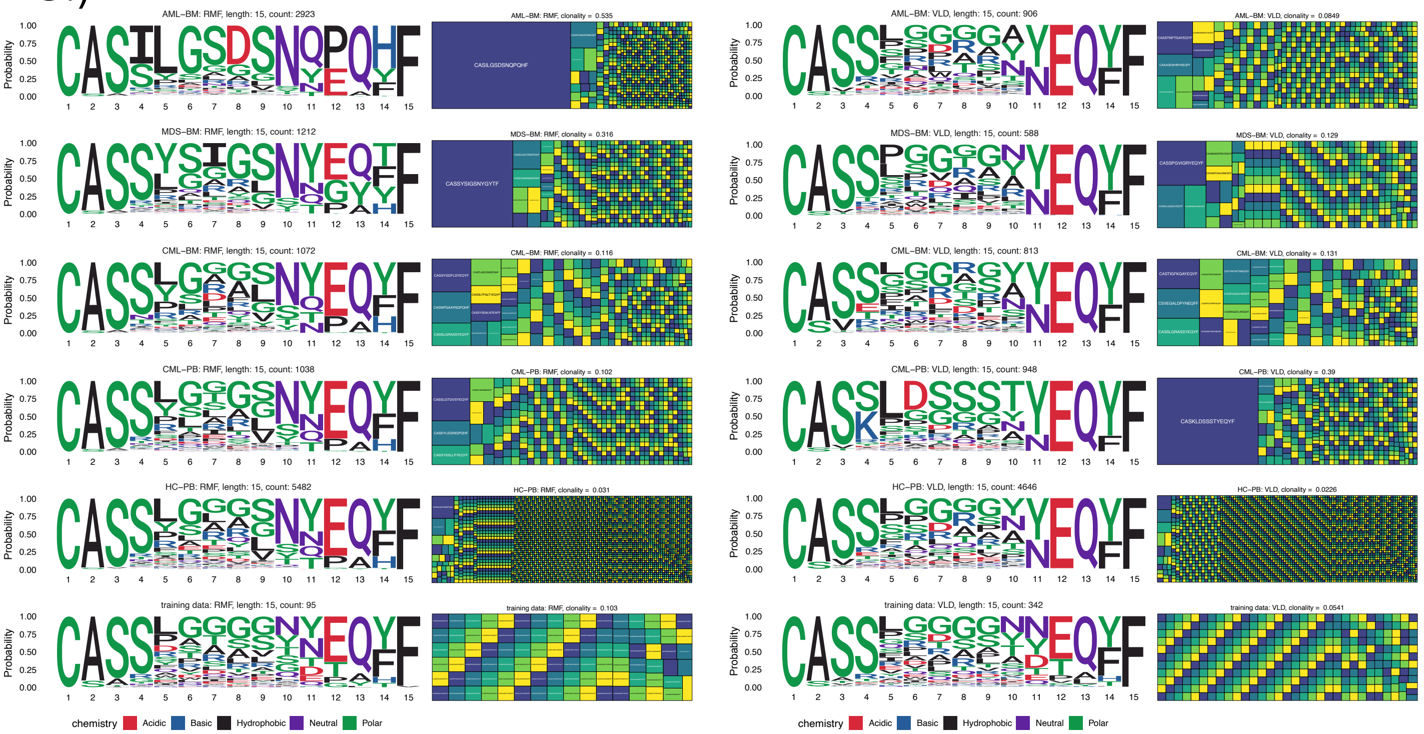

Supplemental Figure 6. CDR3β sequence logos of RMF- and VLD-specific T-cells across different cohorts
A: Distribution of CDR3β sequence lengths across bulk TCRβ cohorts. **B**: Simpson clonality of CDR3β sequences within each bulk TCRβ cohort for RMF-specific, VLD-specific, and total T-cells. **C**: Sequence logos (seqlogos) of all CDR3β sequences of length 15 predicted to recognize RMF or VLD in each cohort and the training dataset. Titles indicate cohort type, sequence length, and the number of sequences used. Treemaps to the right of each seqlogo visualize clonality, with Simpson clonality values included in the title.

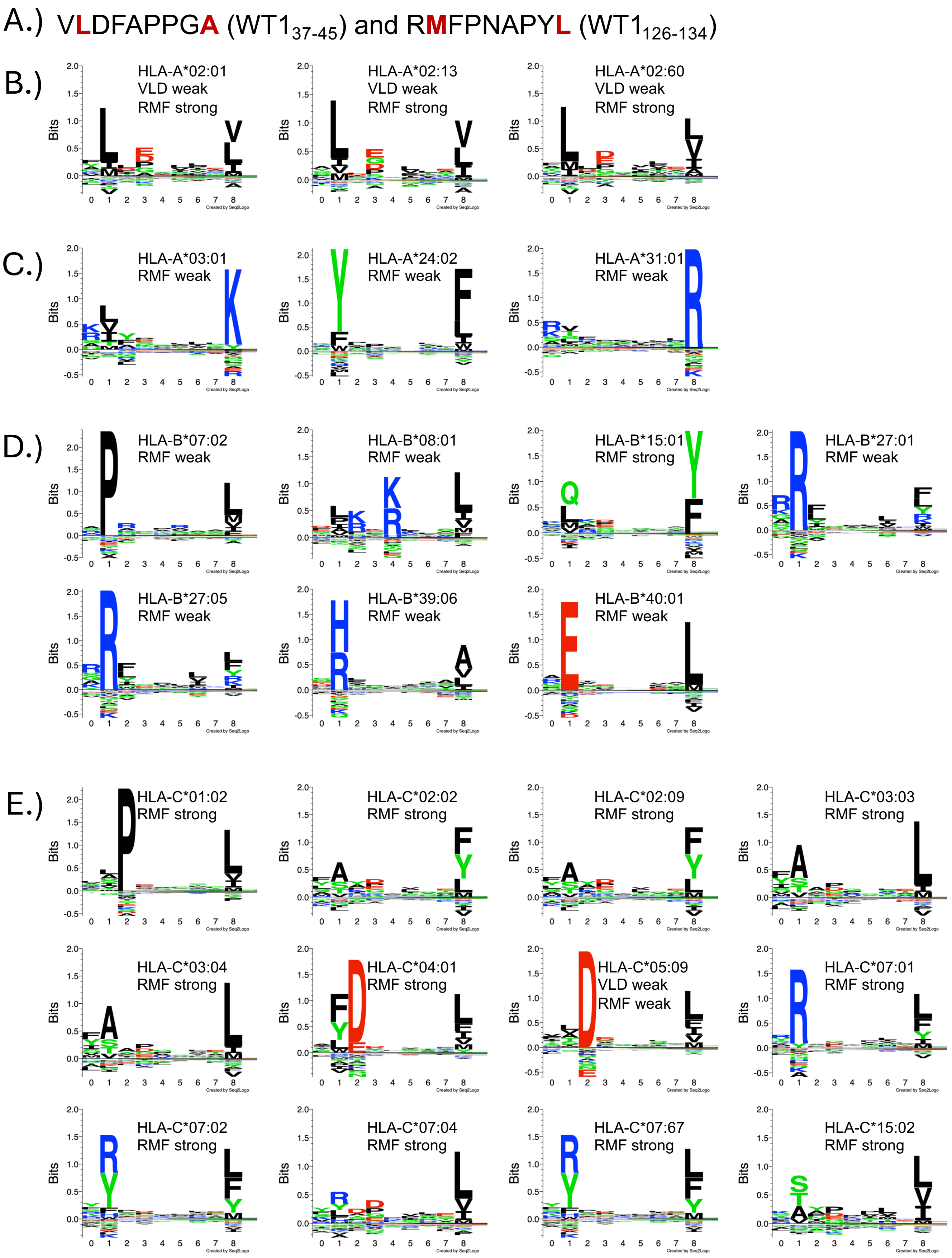

Supplemental Figure 7. HLA-class I allele sequence binding motifs. Legend on the next page.

Supplemental Figure 7. HLA-class I allele sequence binding motifs. WT1 epitope peptide sequences, common anchor residues (amino acids at the position P2 and P9) are highlighted in red (**A**). Sequence logos of HLA-A*02 alleles presenting both WT1 VLD and RMF peptides as natural ligands (**B**). As an example, HLA-A*02:01 motif shows that peptides presented by the allele more frequently have either L (leucine), M (methionine), I (isoleucine) or V (valine) as second residue and V (valine), L (leucine), I (isoleucine), A (alanine) or M (methionine) as ninth residue, in addition fourth residue shows some preference for E (glutamic acid), D (aspartic acid), P (proline), G (glycine) or A (alanine). Sequence logos for HLA-A (**C**) HLA-B (**D**) and HLA-C (**E**) alleles putatively presenting either VLD or RMF peptide. HLA-I sequence binding motifs corresponding AML and CML patient HLA-class I haplotypes are shown, with categorization of epitope binding potential. The amino acid colors refer to their biochemical properties, where black color denotes hydrophobic, green polar, blue positively charged and red negatively charged residues.

Motifs were adopted from NetMHCpan-4.1 Motif Viewer tool at https://services.healthtech.dtu.dk/services/NetMHCpan-4.1/. Peptide categorization as ‘weak’ or ‘strong’ binder is based on peptide binding prediction using NetMHCpan 4.1b (10).

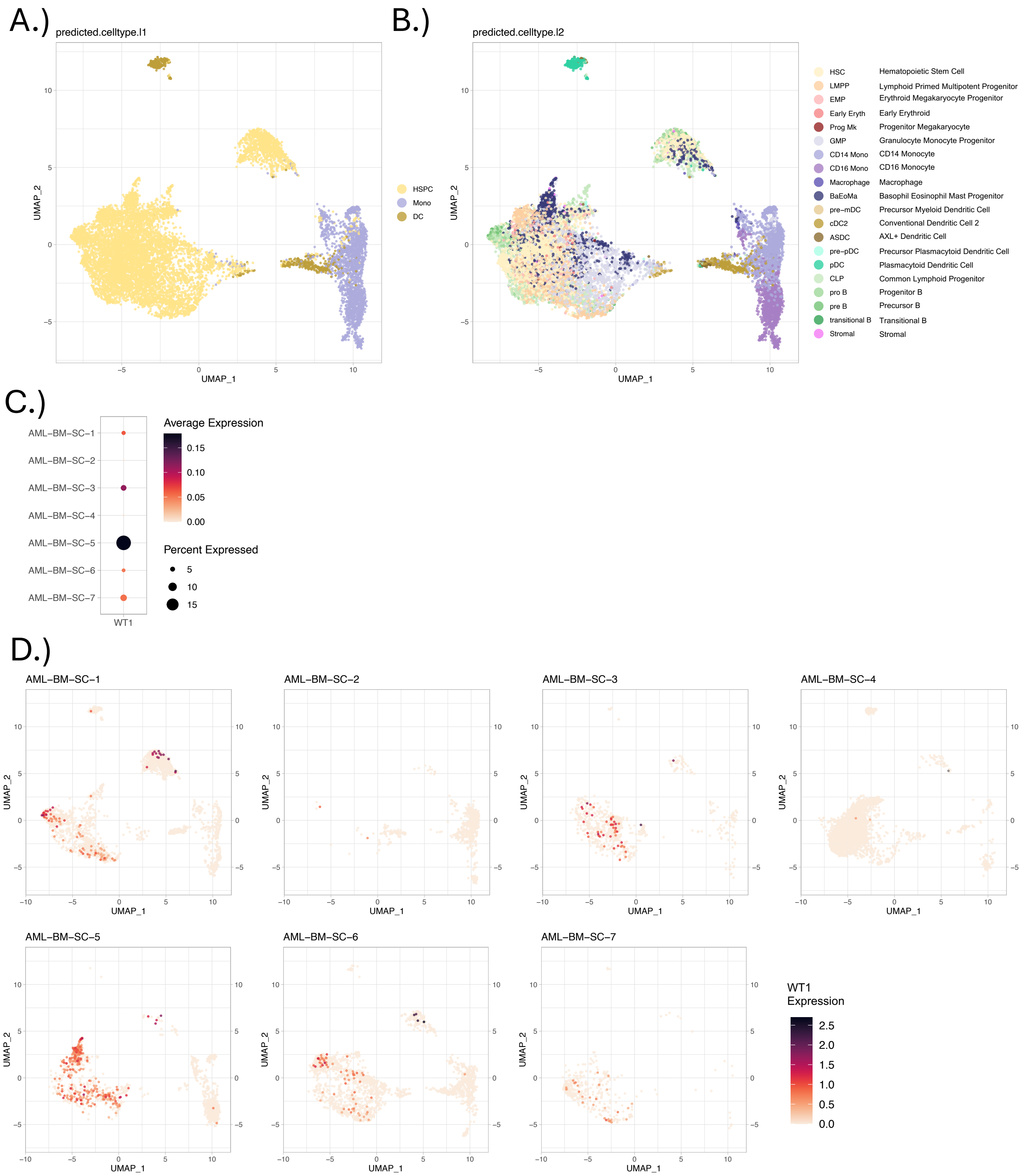

Supplemental Figure 8. WT1 expression of AML-BM-SC cohort patients' HSPCs, monocytes and DCs. UMAP representations of hematopoietic stem and progenitor cells (HSPC), monocytes (Mono) and denritic cells (DC) assinged with Azimuth using its bone marrow reference (**A**), and more refined celltypes of the same cells (**B**). Average expression of *WT1* and percent expressed for each patient is shown in panel (**C**) and UMAP representations of *WT1* expression for each patient separately in panel (**D**).

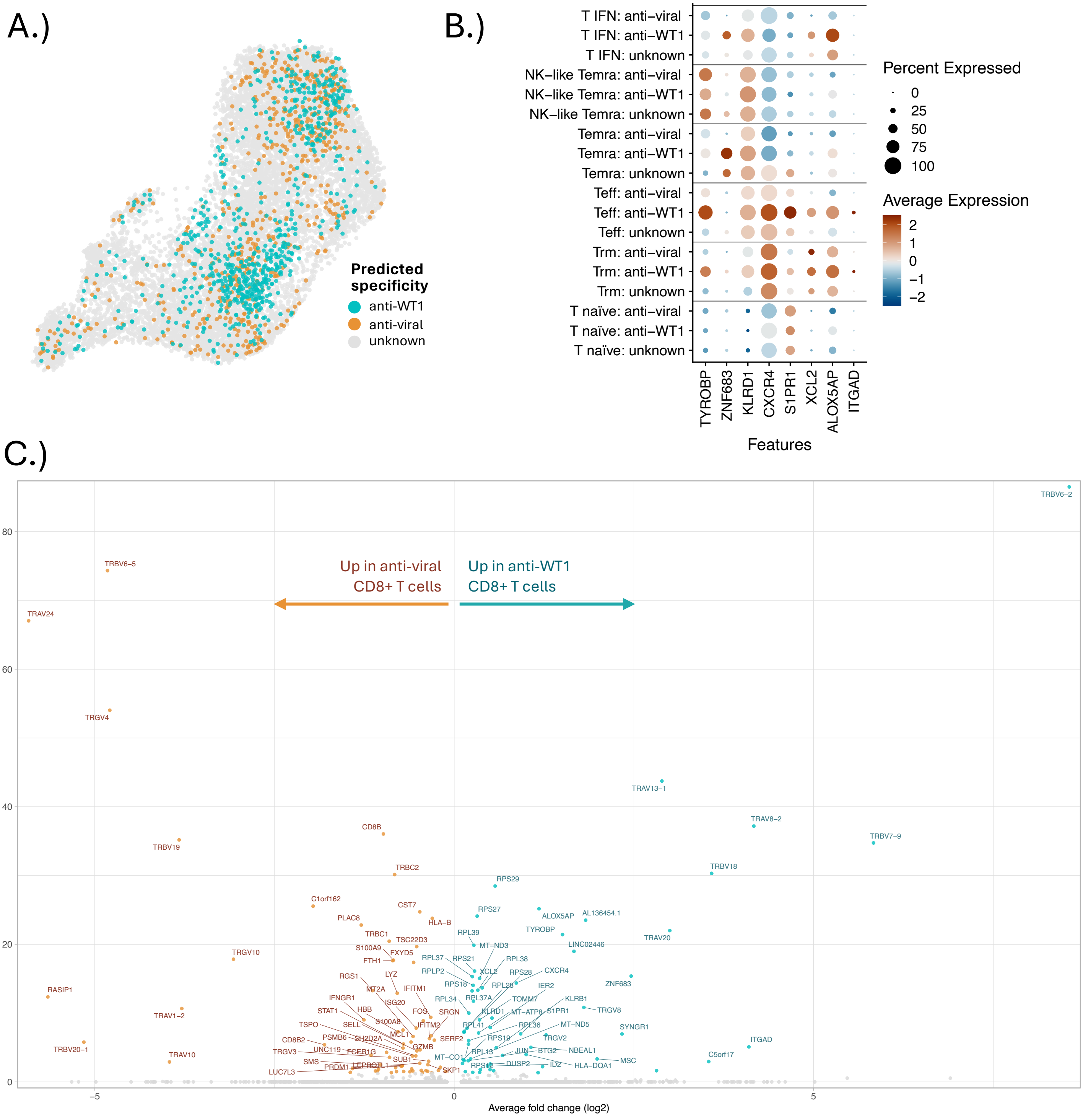

Supplemental Figure 9. Comparison of transcriptomics of anti-WT1 and anti-viral CD8+ T cells in AML-BM-SC cohort.

A: UMAP representations of CD8+ T cells. T cells predicted to be specific to VLD or RMF are marked as anti-WT1, T cells predicted to be specific to CMV epitope NLVPMVATV, IAV epitope GILGFVFTL, EBV epitope GLCTLVAML, or SARS-CoV-s epitope YLQPRTFLL are marked as anti-viral, and T cells not predicted to be in either of the two groups or in both of them are marked as unknown. **B:** Average expression of selected genes upregulated in anti-WT1 T cells compared to anti-viral T cells and percent expressed in T cells with different phenotypes and specificities. **C:** Volcano plot of differentially expressed genes between anti-WT1 and anti-viral T cells.

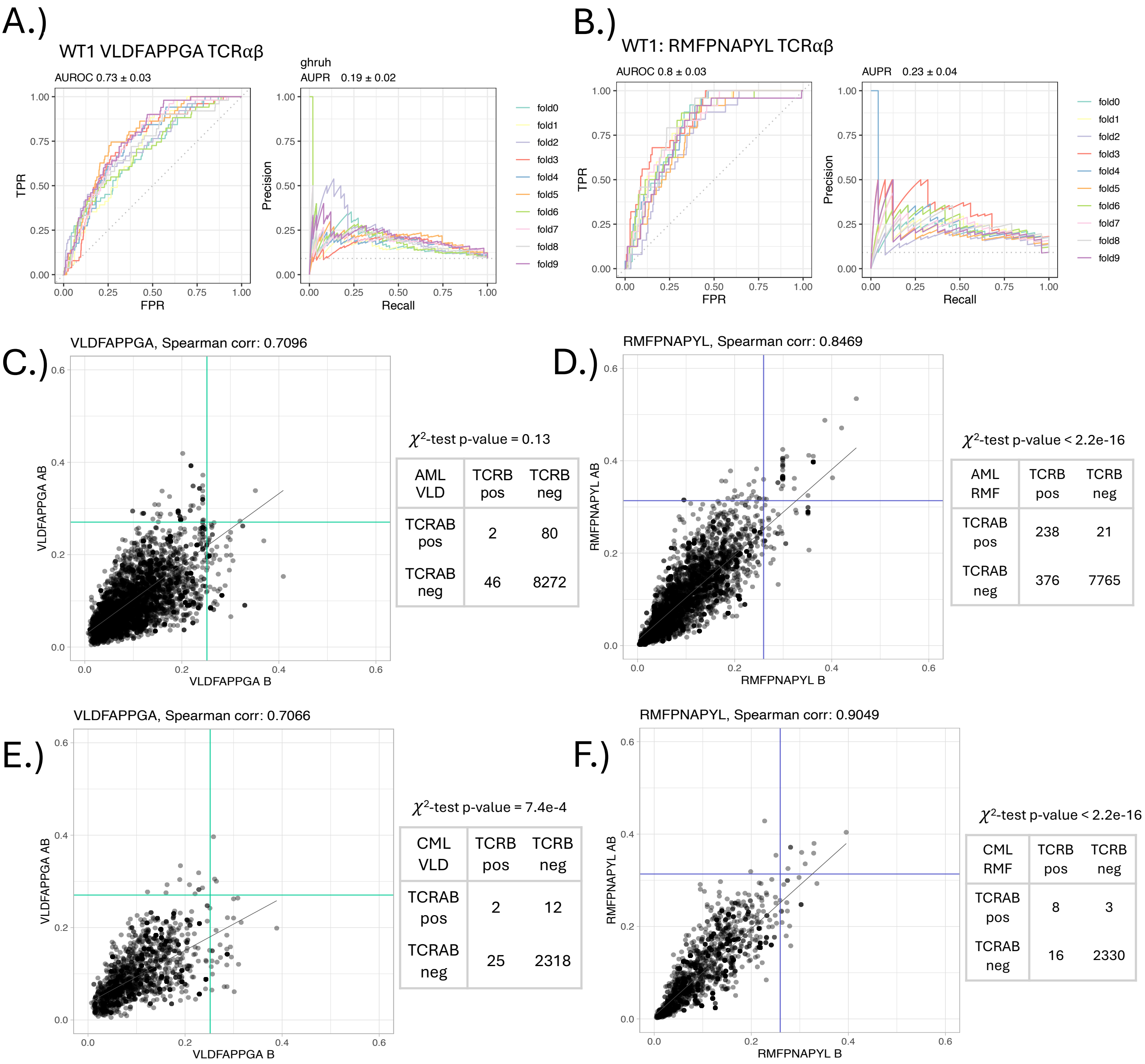

Supplemental Figure 10. Comparison of WT1 TCRGP models trained with TCRβ or TCRαβ. Accuracy of TCRGP models trained with paired TCRαβ data. Models are evaluated as in Supplementary Figure 3: via stratified 10-fold cross-validation using one part epitope-specific TCRs and ten parts non-specific TCRs. AUROC and AUPR scores for VLD are presented in **(A)** and for RMF in **(B)**. Panels **C-D** show the Spearman correlation of TCRGP predictions with TCRβ (on x-axis) and paired TCRαβ (on y-axis) for WT1. The prediction thresholds are marked with colored horizontal and vertical lines and the points above or to the right of them are considered as positive predictions. The line fitted between the predictions is shown as grey line. Additionally, Chi-squared test was used to assess the concordance between the postive and negative predictions. The contingency tables used for the test and its p-value are shown next to the correlation plots. Only T cells with valid TCRα and TCRβ are used in these evaluations. Results for AML-BM-SC dataset are shown in panel **C** for VLD and in panel **(D)** for RMF, and results for CML-PB-SC dataset in **(E)** for VLD and in **(F)** for RMF.

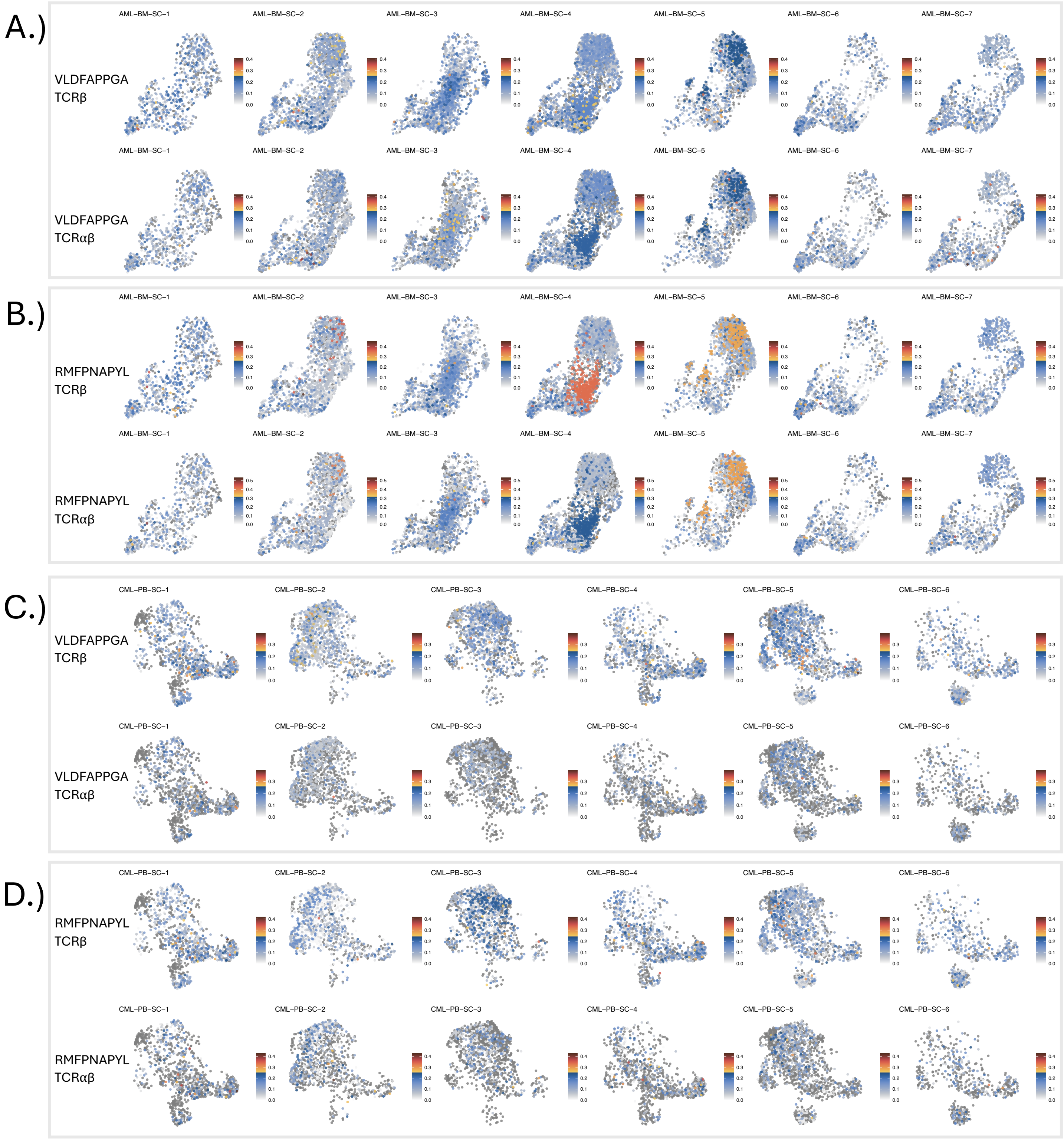

Supplemental Figure 11. Comparison of WT1 TCRGP models trained with TCRβ or TCRαβ.
UMAP representations of scRNA+TCRαβseq data separately for each subject, with each T cell colored based on its TCRGP prediction. The negative predictions are shown in grey-blue scale ranging from zero to the prediction threshold that corresponds to FPR=0.01 while the positive predictions are shown in yellow-red scale ranging from the prediction threshold to the maximum predictive value in the dataset. Cells without a TCR for which a prediction could be made are colored in dark grey. The top row in each panel shows predictions with a TCRGP model using TCRβ and the bottom row with TCRαβ. For AML-BM-SC dataset the predictions for VLD are shown in panel **(A)** and for RMF in panel **(B)**. For CML-PB-SC dataset the predictions for VLD are shown in panel **(C)** and for RMF in panel **(D)**.

Supplemental Table 1. TCR sequences and αβ-pair information from Healthy CD8+ T-cells pulsed with VLD This table contains TCR sequences derived from VLD-pulsed Healthy donor cells isolated via FACS for dextramer positivity. Provided TCR information includes the TCR chain, variable (V) gene, diversity (D) gene, joining (J) gene, constant (C) gene, as well as the amino acid and nucleotide sequences of CDR1, CDR2, and CDR3. Additionally, variable gene allelic data are included.

Supplemental Table 2. TCR sequences and αβ-pair information from AML CD8+ T-cells pulsed with VLD This table contains TCR sequences derived from VLD-pulsed AML patient cells isolated via FACS for dextramer positivity. Provided TCR information includes the TCR chain, variable (V) gene, diversity (D) gene, joining (J) gene, constant (C) gene, as well as the amino acid and nucleotide sequences of CDR1, CDR2, and CDR3. Additionally, variable gene allelic data are included.

Supplemental Table 3. TCR sequences and αβ-pair information from Healthy CD8+ T-cells pulsed with RMF This table contains TCR sequences derived from RMF-pulsed Healthy donor cells isolated via FACS for dextramer positivity. Provided TCR information includes the TCR chain, variable (V) gene, diversity (D) gene, joining (J) gene, constant (C) gene, as well as the amino acid and nucleotide sequences of CDR1, CDR2, and CDR3. Additionally, variable gene allelic data are included.

Supplemental Table 4. TCR sequences and αβ-pair information from AML CD8+ T-cells pulsed with RMF This table contains TCR sequences derived from RMF-pulsed AML patient cells isolated via FACS for dextramer positivity. Provided TCR information includes the TCR chain, variable (V) gene, diversity (D) gene, joining (J) gene, constant (C) gene, as well as the amino acid and nucleotide sequences of CDR1, CDR2, and CDR3. Additionally, variable gene allelic data are included.

Supplemental Table 5. GLIPH2-selected TCR sequences specific to VLD This table contains TCR sequences derived from VLD-pulsed CD8+ T-cells from our healthy control and AML patient donor cohorts, combined with the Gielis-healthy cohort, and selected via GLIPH2 clustering. The table includes CDR3β and TCRβ-chain information.

Supplemental Table 6. GLIPH2-selected TCR sequences specific to RMF This table contains TCR sequences derived from RMF-pulsed CD8+ T-cells from our healthy control and AML patient donor cohorts, combined with the Gielis-healthy cohort, and selected via GLIPH2 clustering. The table includes CDR3β and TCRβ-chain information.

Supplemental Table 7. Patient demagraphic and clinical characteristics for the TCRβ AML-BM cohort This table provides clinical data for patients in the AML-BM cohort. Included information consists of patient cohort ID, sampling time, gender, age, disease risk scores, blast percentage, total TCR count, downsampled TCR count, and the frequency of epitope-specific TCRs for VLDFAPPGA, RMFPNAPYL, NLVPMVATV, GILGFVFTL, GLCTLVAML, YLQPRTFLL, and ELAGIGILTV.

Supplemental Table 8. Patient and sample characteristics for the TCRβ MDS-BM cohort This table provides clinical and sequencing data for patients in the MDS-BM cohort. Data fields include patient cohort ID, sampling time, gender, age, disease risk scores, blast percentage, total TCR count, downsampled TCR count, and the frequency of epitope-specific TCRs for VLDFAPPGA, RMFPNAPYL, NLVPMVATV, GILGFVFTL, GLCTLVAML, YLQPRTFLL, and ELAGIGILTV.

Supplemental Table 9. Patient and sample characteristics for the TCRβ CML-BM cohort This table details clinical and sequencing data for the CML-BM cohort. It includes patient cohort ID, sampling time, gender, age, disease risk scores, blast percentage, total TCR count, downsampled TCR count, and the frequency of epitope-specific TCRs for VLDFAPPGA, RMFPNAPYL, NLVPMVATV, GILGFVFTL, GLCTLVAML, YLQPRTFLL, and ELAGIGILTV.

Supplemental Table 10. Patient and sample characteristics for the TCRβ CML-PB cohort This table presents clinical and sequencing data for the CML-PB cohort, including patient cohort ID, sampling time, gender, age, disease risk scores, blast percentage, total TCR count, downsampled TCR count, and the frequency of epitope-specific TCRs for VLDFAPPGA, RMFPNAPYL, NLVPMVATV, GILGFVFTL, GLCTLVAML, YLQPRTFLL, and ELAGIGILTV.

Supplemental Table 11. Patient and sample characteristics for the TCRβ HC-PB cohort This table provides clinical and sequencing data for the HC-PB healthy cohort. Included fields are patient cohort ID, sampling time, gender, age, CMV status, HLA alleles, total TCR count, downsampled TCR count, and the frequency of epitope-specific TCRs for VLDFAPPGA, RMFPNAPYL, NLVPMVATV, GILGFVFTL, GLCTLVAML, YLQPRTFLL, and ELAGIGILTV.

Supplemental Table 12. Patient demagraphic and clinical characteristics for the TCRαβ AML-BM-SC cohort This table provides clinical data for patients in the AML-BM-SC cohort. Included information consists of patient cohort ID, sampling time, gender, age, disease risk scores, blast percentage, HLA alleles, CMV and EBV status, total TCR count, and the frequency of epitope-specific TCRs for VLDFAPPGA, RMFPNAPYL, NLVPMVATV, GILGFVFTL, GLCTLVAML, YLQPRTFLL, and ELAGIGILTV from whole T-cell compartment and CD8+ T-cells.

Supplemental Table 13. Patient demagraphic and clinical characteristics for the TCRαβ CML-PB-SC cohort This table provides clinical data for patients in the CML-PB-SC cohort. Included information consists of patient cohort ID, sampling time, gender, age, disease risk scores, blast percentage, HLA alleles, CMV and EBV status, total TCR count, and the frequency of epitope-specific TCRs for VLDFAPPGA, RMFPNAPYL, NLVPMVATV, GILGFVFTL, GLCTLVAML, YLQPRTFLL, and ELAGIGILTV from whole T-cell compartment and CD8+ T-cells.

Supplemental Table 14. Statistical testing of patient cohort differences with respect to healthy cohort.
One-sided Mann-Whitney U-test was used to assess whether **A** the frequencies and **B** clonalities of epitope-specific TCRs in each patient cohort with bulk TCRβ sequencing were higher compared to the healthy cohort (HC-PB). Tables show the Mann-Whitney U statistic, original p-value for each test and p-values adjusted for multiple testing with Benjamini-Hochberg correction.

A.)

Cohort	Epitope	U-statistic	p	p.adj
AML-BM	VLD	341	0.8862	0.8862
MDS-BM	VLD	427	0.8392	0.8862
CML-BM	VLD	243	0.7704	0.8862
CML-PB	VLD	214	0.7175	0.8862
AML-BM	RMF	518	0.0694	0.1388
MDS-BM	RMF	509	0.4544	0.4544
CML-BM	RMF	361	0.0560	0.1388
CML-PB	RMF	284	0.1724	0.2299

B.)

Cohort	Epitope	U statistic	p	p.adj
AML-BM	VLD	754	2.07E-07	4.14E-07
MDS-BM	VLD	880	1.55E-07	4.14E-07
CML-BM	VLD	504	5.13E-06	6.84E-06
CML-PB	VLD	411	1.07E-04	1.07E-04
AML-BM	RMF	757	1.63E-07	4.14E-07
MDS-BM	RMF	877	1.92E-07	4.14E-07
CML-BM	RMF	516	1.67E-06	2.67E-06
CML-PB	RMF	414	8.22E-05	9.39E-05

Supplemental Table 15. Predicted HLA class I binding to VLD and RMF in AML-BM-SC and CML-PB-SC cohorts.
The HLA class I alleles were predicted with Optitype (10) and their binding to VLD and RMF were predicted with NetMHCpan 4.1b (9). Descriptions of the abbreviations and colors used in the table are given below.

	VLDFAPPGA						RMFPNAPYL					
MHC	Score_EL	%Rank_EL	Score_BA	%Rank_BA	Aff(nM)	BindLevel	Score_EL	%Rank_EL	Score_BA	%Rank_BA	Aff(nM)	BindLevel
HLA-A*01:01	0.023154	3.09	0.162568	2.567	8611.29		0.009278	5.075	0.074443	11.668	22344.19	
**HLA-A*02:01**	0.327768	0.686	0.480222	1.996	276.96	WB	0.954738	0.021	0.841136	0.05	5.58	SB
**HLA-A*02:13**	0.291052	0.598	0.467879	1.565	316.53	WB	0.899302	0.023	0.786409	0.051	10.08	SB
**HLA-A*02:60**	0.412203	0.534	0.416213	1.898	553.6	WB	0.938838	0.027	0.77738	0.061	11.12	SB
**HLA-A*03:01**	0.001844	10.963	0.085024	16.172	19927.13		0.161109	1.419	0.35658	1.773	1055.39	WB
HLA-A*11:01	0.000177	20.76	0.057781	24.777	26758.32		0.012319	4.767	0.213274	5.32	4975.1	
**HLA-A*24:02**	0.000118	24.786	0.030224	35.564	36053.57		0.289284	0.565	0.395654	0.767	691.52	WB
**HLA-A*31:01**	0.000316	28.3	0.050978	44.915	28802.21		0.243219	1.213	0.514681	1.284	190.77	WB
HLA-A*68:01	0.000185	30.632	0.040029	44.914	32424.63		0.0026	13.039	0.10053	17.848	16849.32	
**HLA-B*07:02**	0.000473	21.041	0.040163	37.934	32377.65		0.064228	1.922	0.222385	3.255	4508.06	WB
**HLA-B*08:01**	0.001051	24.245	0.033759	68.256	34700.64		0.083215	1.922	0.21052	4.933	5125.58	WB
**HLA-B*15:01**	0.000523	23.672	0.048745	50.249	29506.58		0.5335	0.41	0.551067	0.683	128.68	SB
**HLA-B*27:01**	0.000069	35.5	0.008946	65.074	45387.16		0.119372	1.473	0.120058	1.74	13640.21	WB
**HLA-B*27:05**	0.000273	23.345	0.035202	57.845	34163.06		0.144858	1.462	0.318359	2.527	1595.92	WB
HLA-B*35:01	0.00016	26.684	0.033641	36.265	34744.98		0.025296	3.403	0.220308	3.045	4610.51	
HLA-B*35:03	0.0002	21.269	0.022657	24.708	39129.61		0.03924	2.006	0.148832	1.667	9991.09	
HLA-B*35:08	0.000577	16.084	0.040838	24.473	32142.04		0.007958	5.019	0.143135	3.786	10626.32	
**HLA-B*39:06**	0.008235	9.799	0.066622	17.202	24317.29		0.102061	1.878	0.169138	3.369	8020.39	WB
**HLA-B*40:01**	0.000133	22	0.040848	24.058	32138.56		0.045805	1.88	0.249671	1.809	3355.63	WB
HLA-B*40:02	0.000105	31.111	0.031265	48.006	35649.76		0.05328	2.581	0.281843	2.618	2369.18	
HLA-B*44:02	0.000061	34.5	0.025561	63.001	37919.25		0.002435	7.001	0.087714	9.717	19355.5	
HLA-B*44:03	0.000042	37.333	0.018707	68.953	40838.19		0.004976	5.256	0.09653	7.304	17594.56	
HLA-B*51:01	0.000847	26.385	0.02642	36.783	37568.45		0.056181	2.957	0.121328	3.459	13454.06	
HLA-B*56:01	0.004487	9.128	0.04386	15.573	31108.07		0.025905	3.735	0.099726	4.692	16996.54	
**HLA-C*01:02**	0.002588	9.023	0.065432	11.56	24632.41		0.488968	0.139	0.502177	0.091	218.4	SB
**HLA-C*02:02**	0.000136	27.857	0.04568	31.92	30501.49		0.367758	0.245	0.443827	0.277	410.62	SB
**HLA-C*02:09**	0.000136	27.857	0.04568	31.92	30501.49		0.367758	0.245	0.443827	0.277	410.62	SB
**HLA-C*03:03**	0.000127	18.19	0.049355	21.424	29312.46		0.380128	0.303	0.612475	0.258	66.22	SB
**HLA-C*03:04**	0.000127	18.19	0.049355	21.424	29312.46		0.380128	0.303	0.612475	0.258	66.22	SB
**HLA-C*04:01**	0.008271	4.084	0.079531	6.941	21147.36		0.193846	0.316	0.26493	0.117	2844.93	SB
**HLA-C*05:09**	0.054772	1.583	0.22213	1.476	4520.51	WB	0.22891	0.554	0.321251	0.619	1546.75	WB
**HLA-C*07:01**	0.000061	27.833	0.048074	31.148	29721.57		0.129662	0.242	0.427948	0.157	487.59	SB
**HLA-C*07:02**	0.000175	20.609	0.0457	32.4	30494.89		0.294034	0.176	0.520998	0.09	178.16	SB
**HLA-C*07:04**	0.002227	7.1	0.068649	10.332	23789.78		0.193949	0.081	0.317555	0.033	1609.86	SB
**HLA-C*07:67**	0.000175	20.609	0.0457	32.4	30494.89		0.294034	0.176	0.520998	0.09	178.16	SB
**HLA-C*15:02**	0.000863	16.966	0.076542	17.688	21842.45		0.485373	0.233	0.499816	0.236	224.05	SB

abbreviation	description
BA	binding affinity
EL	(Mass-spectrometry) eluted ligands
Score	The raw prediction score
%Rank	Rank of the predicted binding score compared to a set of random natural peptides. This measure is not affected by inherent bias of certain molecules towards higher or lower mean predicted affinities.
BindLevel	The peptide will be identified as a strong binder (SB) if the %Rank is below 0.5%. The peptide will be identified as a weak binder (WB) if the %Rank is above above 0.5% but below 2%.
**dark green**	**strong RMF and weak VLD binder**
**light green**	**strong RMF binder**
**yellow**	**weak RMF and weak VLD binder**
**light blue**	**weak RMF binder**

Supplemental Table 16. Statistical testing of patient cohort differences in WT1 epitope enrichment per scRNAseq cluster
Enrichment of T cells predicted to be specific to VLD or RMF for each cluster was tested with one-sided Fisher's exact test and p-values adjusted with Benjamini-Hochberg procedure in **A** AML-BM-SC and **B** CML-PB-SC cohorts**.** Only cells with TCR β -chain that a prediction could be made are included. The tables show the original p-values, odds ratio, adjusted p-values and the counts used for computing the tests.

A.)

Hypothesis for AML-BM-SC cohort	p	Odds ratio	p.adj	VLD cells in the cluster	VLD cells in other clusters	Other cells in the cluster	Other cells in other clusters
VLD enriched to cluster Tn	7.59E-03	1.81	3.03E-02	26	94	1320	8661
VLD enriched to cluster Tem/rm	1.13E-03	1.91	6.77E-03	38	82	1946	8035
VLD enriched to cluster Teff	8.05E-01	0.84	1.00E+00	26	94	2462	7519
VLD enriched to cluster Temra	9.87E-01	0.64	1.00E+00	27	93	3122	6859
VLD enriched to cluster NK-like Temra	1.00E+00	0.16	1.00E+00	2	118	939	9042
VLD enriched to cluster IFN CTL	9.03E-01	0.43	1.00E+00	1	119	192	9789
				**RMF cells in the cluster**	**RMF cells in other clusters**	**Other cells in the cluster**	**Other cells in other clusters**
RMF enriched to cluster Tn	1.00E+00	0.15	1.00E+00	16	649	1330	8106
RMF enriched to cluster Tem/rm	9.96E-01	0.75	1.00E+00	105	560	1879	7557
RMF enriched to cluster Teff	1.74E-32	2.70	2.09E-31	299	366	2189	7247
RMF enriched to cluster Temra	5.95E-01	0.98	1.00E+00	205	460	2944	6492
RMF enriched to cluster NK-like Temra	1.00E+00	0.32	1.00E+00	22	643	919	8517
RMF enriched to cluster IFN CTL	8.48E-02	1.47	2.54E-01	18	647	175	9261

B.)

Hypothesis for CML-PB-SC cohort	p	Odds ratio	p.adj	VLD cells in the cluster	VLD cells in other clusters	Other cells in the cluster	Other cells in other clusters
VLD enriched to cluster Tn	2.54E-02	1.99	8.48E-02	13	65	455	4524
VLD enriched to cluster Tem/rm	5.73E-07	3.19	5.73E-06	40	38	1235	3744
VLD enriched to cluster NK-like Temra	1.00E+00	0.31	1.00E+00	19	59	2521	2458
VLD enriched to cluster IFN CTL	9.34E-01	0.50	1.00E+00	3	75	369	4610
VLD enriched to cluster MAIT-like	9.54E-01	0.46	1.00E+00	3	75	399	4580
				**RMF cells in the cluster**	**RMF cells in other clusters**	**Other cells in the cluster**	**Other cells in other clusters**
RMF enriched to cluster Tn	6.86E-03	2.84	3.43E-02	10	35	458	4554
RMF enriched to cluster Tem/rm	7.94E-02	1.64	1.98E-01	16	29	1259	3753
RMF enriched to cluster NK-like Temra	9.93E-01	0.49	1.00E+00	15	30	2525	2487
RMF enriched to cluster IFN CTL	9.68E-01	0.28	1.00E+00	1	44	371	4641
RMF enriched to cluster MAIT-like	7.06E-01	0.83	1.00E+00	3	42	399	4613

Supplemental Table 17. iCAN consortium. List of all members of the iCAN consortium.
